# Oral splints for temporomandibular disorder or bruxism: a systematic review

**DOI:** 10.1038/s41415-020-1250-2

**Published:** 2020-02-14

**Authors:** Philip Riley, Anne-Marie Glenny, Helen V. Worthington, Elisabet Jacobsen, Clare Robertson, Justin Durham, Stephen Davies, Helen Petersen, Dwayne Boyers

**Affiliations:** 495990657843294236023 Cochrane Oral Health, Division of Dentistry, School of Medical Sciences, The University of Manchester, UK; 891834382857393586459 Health Economics Research Unit, University of Aberdeen, UK; 112095844478012536480 Health Services Research Unit, University of Aberdeen, UK; 234842423445644238676 Centre for Oral Health Research & School of Dental Sciences, Newcastle University, UK; 157954082765753835179 Division of Dentistry, School of Medical Sciences, The University of Manchester, UK

## Abstract

**Objectives** To evaluate the clinical-effectiveness of oral splints for patients with TMD or bruxism for the primary outcomes: pain (TMD) and tooth wear (bruxism).

**Data sources ** Four databases including MEDLINE and EMBASE were searched from inception until 1 October 2018.

**Data selection and extraction** Randomised controlled trials comparing all types of splints versus no/minimal treatment for patients with TMD or bruxism were eligible. Standard Cochrane review methods were used. Standardised mean differences (SMD) were pooled for the primary outcome of pain, using random effects models in TMD patients.

**Data synthesis** Thirty-seven trials were included and the evidence identified was of very low certainty using GRADE assessments. When all subtypes of TMD were pooled into one global TMD group, there was no evidence that splints reduced pain: SMD (up to 3 months) -0.18 (95% CI -0.42 to 0.06); 13 trials, 1,076 participants. There was no evidence that any other outcomes improved when using splints. There was no evidence of adverse events associated with splints, but reporting was poor regarding this outcome. No trials measured tooth wear in patients with bruxism. There was a large variation in diagnostic criteria, splint types and outcome measures used and reported. Sensitivity analyses based on these factors did not indicate a reduction in pain.

**Conclusions** The very low-certainty evidence identified did not demonstrate that splints reduced pain in TMD as a group of conditions. There is insufficient evidence to determine whether splints reduce tooth wear in patients with bruxism.

Zusatzmaterial online: Zu diesem Beitrag sind unter 10.1038/s41415-020-1250-2 für autorisierte Leser zusätzliche Dateien abrufbar.

## Key points

- This systematic review comprehensively summarised the best available evidence from randomised controlled trials on the effects of oral splints for temporomandibular disorders (TMD) and bruxism.
- There is no evidence to support the use of oral splints for either condition based on the results found.
- For TMD patients, sensitivity analyses were conducted to explore the effects of differences in: 1) diagnostic criteria; 2) splint types; and 3) outcome measures used and reported. There were no differences in the results based on these factors.

## Introduction

Temporomandibular disorders (TMD) are the second most common cause (after dental pain) of orofacial pain, characterised by pain in the temporomandibular joint area and in the facial muscles. Apart from pain, patients may experience other signs and symptoms, such as clicking of the joint and restricted mouth-opening. Around 5% to 12% of the population have TMD symptoms to some degree, varying by age group and gender.^1^ One of the most common ways in which dentists, particularly in primary care, manage symptomatic TMD is the provision of oral splints.^2^

Splints are also provided to help manage tooth wear caused by bruxism. The prevalence of bruxism ranges from 8% to 31% within the general population,^3^ and it is estimated globally that sleep bruxism affects 16%, and awake bruxism 24%, of the adult population.^4^

There is continuing debate about the exact mechanism of action of oral splints. Mechanisms include: muscle relaxation/habit-breaking for patients with increased parafunctional or muscle-tightening habits; protection of teeth and jaws, particularly where teeth clenching and grinding may lead to damage of teeth; normalising periodontal ligament proprioception, by utilising a splint to spread the forces placed on individual teeth; and repositioning of the jaws and condyles into centric relation.

This systematic review arose from an National Institute for Health Research Health Technology Assessment call addressing the research question: 'What is the clinical and cost-effectiveness of prefabricated oral splints and custom-made splints for the treatment of orofacial symptoms?' (in press). This research presents part of this review, looking at whether oral splints are effective in reducing orofacial symptoms (primarily pain) and when they are indicated to prevent tooth wear.

## Methods

We undertook the review using Cochrane methods,^5^ which are described in greater detail elsewhere (in press).

### Eligibility criteria

Randomised controlled trials were included (crossover studies were excluded as deemed inappropriate). We included children (over 11 years old) and adults who had either TMD or bruxism, in either primary or secondary care.

We included trials where any type of splint was compared with a non-splint group. This group also included watchful waiting or minimal treatment (advice/counselling, education or self-performed exercises).

The primary outcomes were pain and harms. For bruxism patients, tooth wear was also considered a primary outcome. Secondary outcomes included clicking of the temporomandibular joint, change in restricted mouth opening, frequency of headaches and reduced quality of life. Patient satisfaction and adherence to treatment were collected whenever possible. For bruxism, the index and frequency of bruxism activity was also to be recorded.

Follow-up periods for the outcome data were divided into short-term follow-up (0 to 3 months), medium-term (>3 to 6 months), or long-term (>6 to 12 months).

### Search methods for identification of studies

An information specialist developed a search strategy (see online-only Supplementary Appendix 1) and conducted the literature searches on 1 October 2018. They were undertaken without restrictions on language or date of publication.

The following databases were searched: Cochrane Central Register of Controlled Trials (CENTRAL) in the Cochrane Library, MEDLINE Ovid, Embase Ovid, and CINAHL EBSCO. Unpublished data were sought via searches of the US National Institutes of Health trials register (ClinicalTrials.gov) and the WHO International Clinical Trials Registry Platform. Conference proceedings were searched via Embase and the Web of Science. Abstracts of dissertations and theses were searched via the Proquest database. Additional grey literature was sourced through the American Academy of Dental Sleep Medicine (AADSM; http://www.aadsm.org/) website. The International Association of Dental Research (IADR) annual conference abstracts were searched via the IADR website.

### Selection of studies and data extraction

Two review authors independently assessed studies retrieved by the searches for eligibility. Disagreements on study eligibility were resolved through discussion and consensus. If necessary, a third review author was consulted. Two review authors independently extracted the following data from the included trials: location/setting, type of provider, number of centres, recruitment period, trials registry ID, inclusion/exclusion criteria, demographic information, presenting condition and severity, type of splint, details of comparator, outcomes reported (including method and time of assessment), details of sample size calculations, funding sources and declarations/conflicts of interest.

### Assessment of risk of bias in included studies

The assessment of risk of bias was done independently and in duplicate, using the Cochrane risk of bias tool.^5^ The following domains were assessed: sequence generation; allocation concealment; blinding of participants and personnel; blinding of outcome assessors; incomplete outcome data; selective outcome reporting; and other bias. The overall risk of bias of individual studies was categorised as: low (plausible bias unlikely to seriously alter the results), if all domains were at low risk of bias; unclear (plausible bias that raises some doubt about the results), if one or more domains had an unclear risk of bias; or high, if one or more domains had a high risk of bias.

### Statistical methods

For continuous outcomes, we used the means and standard deviations reported in the trials to express the estimate of effect as mean difference with 95% confidence interval (CI). If different scales were reported, we used standardised mean difference (SMD). For dichotomous outcomes, we expressed the estimate of effect as a risk ratio with 95% CI.

We attempted to contact the author(s) of all included studies, where feasible, in the event of missing data. Missing standard deviations were estimated.^5^

We assessed heterogeneity statistically by using a Chi² test, where a P value of less than 0.1 indicates statistically significant heterogeneity. We quantified heterogeneity by using the I² statistic.

We combined mean differences (or SMDs) for continuous data, and risk ratios for dichotomous data, using random effects models.

### Sensitivity analysis

For TMD patients, we undertook a sensitivity analysis restricted to trials where the inclusion criteria were based on, or could be clearly mapped to, one of the following sets of diagnostic criteria: Research Diagnostic Criteria for Temporomandibular disorders (RDC/TMD) guidelines;^6^ TMD (DC/TMD) guidelines;^7^and American Association of Orofacial Pain (AAOP) guidelines.^8^ The outcome measures used and reported varied between studies; therefore, a sensitivity analysis was also carried out including only studies that measured pain at the time of assessment (current pain) on a 0 to 100 visual analogue or numerical rating scale. We also carried out sensitivity analysis based on splint types, restricting the analyses to studies using stabilisation splints.

Similarly, for bruxism patients, we planned to undertake a sensitivity analysis restricted to trials with a clear diagnosis of bruxism.^9^ The study should have used polysomnography (PSG) to diagnose the bruxism. There were insufficient trials to do this.

### Presentation of main results

The certainty of the body of evidence was assessed following GRADE methods,^10^ considering the overall risk of bias of included studies, the directness of the evidence, the inconsistency of the results, the precision of the estimates and the risk of publication bias. We categorised the certainty of the body of evidence for each of the main outcomes for each comparison as high, moderate, low or very low.

## Results

### Characteristics of included studies

**Fig. 1:**
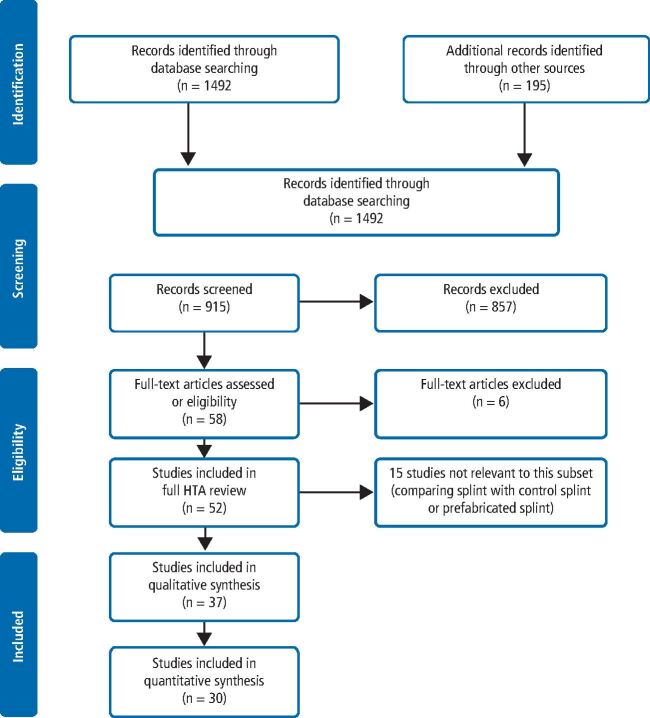
Flow of studies through the review process

Thirsty-seven studies were included (Fig. 1); 34 on patients with TMD and two on patients with bruxism, with a further study on patients with both TMD and bruxism. All studies, with the exception of one, were conducted in universities or public hospitals/clinics.

For the studies evaluating the effectiveness of splints for people with TMD, the diagnostic criteria for TMD varied. However, the predominantly used criteria were the RDC,^6^ used in 17 studies.^11^^,^^12^^,^^13^^,^^14^^,^^15^^,^^16^^,^^17^^,^^18^^,^^19^^,^^20^^,^^21^^,^^22^^,^^23^^,^^24^^,^^25^^,^^26^^,^^27^ The DC criteria^7^ were used in two studies,^28^^,^^29^ and an additional three studies used criteria that approximated to the RDC (either by citing the instrument and/or their description matched a similar process).^30^^,^^31^^,^^32^ No studies used the AAOP criteria.^8^

The remaining studies used criteria that we had not pre-specified in our protocol or that were undefined/unclear:

- Three had used the Helkimo index^33^^,^^34^^,^^35^
- Two used arthrography^36^^,^^37^
- One used MRI^38^
- Six used diagnostic systems that were not possible to classify.^39^^,^^40^^,^^41^^,^^42^^,^^43^^,^^44^

The two studies^45^^,^^46^ examining the effects of splints on bruxism used the Lobbezzo *et al*.^9^ criteria for likelihood of a bruxism diagnosis: 'possible' self-report of bruxism; 'probable' clinical evidence of bruxism with or without self-report; and 'definite' defined by polysomnography. We classified both studies as examining 'probable' bruxism.

The study that examined bruxism with co-morbid TMD used the Fonseca index for TMD and examined 'probable' bruxism.^47^

Thirty-five studies compared splints against no splints for TMD patients. Ten of these studies used a no treatment control group.^14^^,^^23^^,^^30^^,^^32^^,^^33^^,^^34^^,^^35^^,^^37^^,^^41^^,^^43^ Twenty had a co-intervention in each arm, with 13 having a 'minimal' co-intervention of usual treatment, counselling, information or exercise,^11^^,^^12^^,^^13^^,^^15^^,^^16^^,^^17^^,^^18^^,^^19^^,^^21^^,^^22^^,^^25^^,^^26^^,^^36^ while 7 had a 'non-minimal' co-intervention of 'acuhealth', manipulative and physical therapy, massage, Prozac, microcurrent electrical nerve stimulation, physical therapy with vapocoolant spray, arthrocentesis and sodium hyaluronate.^27^^,^^28^^,^^29^^,^^39^^,^^40^^,^^44^^,^^47^ The remaining six studies had minimal treatment controls: three were self-exercises,^24^^,^^28^^,^^42^ and three were information-based.^20^^,^^27^^,^^31^

One trial that has been referenced twice above^27^ had four arms with which we made two separate pairwise comparisons: 1) splint + co-intervention vs co-intervention alone; and 2) splint vs minimal treatment.

Nineteen studies used a stabilisation splint, 14 of which were in the upper jaw (Michigan-style splints),^11^^,^^13^^,^^14^^,^^19^^,^^20^^,^^21^^,^^26^^,^^27^^,^^35^^,^^38^^,^^40^^,^^42^^,^^45^^,^^47^ but not clearly reported in the other five.^16^^,^^22^^,^^24^^,^^29^^,^^30^

Seven studies compared more than one splint against no splint in this comparison, and were included twice in any meta-analysis as two separate pairwise comparisons.

For more details on the characteristics of the included studies, see online-only Supplementary Appendix 2.

### Risk of bias

All 37 studies were assessed as being at high risk of bias overall due to a rating of high risk for at least one of the seven domains assessed (see Risk of bias summary in online-only Supplementary Appendix 3).

Ten studies were at low risk of selection bias because they adequately described methods used both to generate a truly random sequence and also to conceal the sequence from those involved in the randomisation process. The remaining studies were at unclear risk of selection bias as they had an unclear rating for either random sequence generation, allocation concealment, or both.

All studies, unavoidably, were at high risk of performance bias due to the comparison of splint against no splint. Thirty-five studies were at high risk of detection bias because patients knew their assigned group and also subjectively rated their own pain. The remaining two studies had a low risk of detection bias, the first being due to objective assessment of bruxism while the patients slept,^46^ and the second because no outcomes of this review were assessed and therefore this bias domain was irrelevant.^47^

Nine studies were at high risk of attrition bias due to high rates of attrition, large differences in attrition rates between groups, or both. The remaining six studies had an unclear risk of attrition bias due to poor reporting of numbers randomised or analysed.

Sixteen studies had problems with the way in which data were reported and were at high risk of reporting bias.

For other potential sources of bias, three studies were given a rating of high risk because outcomes were followed up at different times for the two groups. A further three studies were given an unclear rating for this domain because the reporting was poor and we were unable to properly assess them.

For more details on the risk of bias of the included studies, see online-only Supplementary Appendix 2.

### Patients with TMD

There was consensus with clinicians and methodologists that 0 to 3 months was an appropriate time point to use for the primary analysis of the data. The primary pain outcome was any continuous scale that was sensible to combine (for example, Visual Analogue Scale (VAS), Numerical Rating Scale (NRS), Characteristic Pain Intensity (CPI)).

Thirteen trials of 16 pairwise comparisons, all at high risk of bias and with 1,076 patients, contributed to the results for the main comparison at three months (Table 1). There was considerable heterogeneity and the overall SMD was -0.18 (95% CI -0.42 to 0.06). Using a rule of thumb for SMD effect estimates,^5^ 0.18 would be considered a small effect and, as this was not statistically significant, there is no evidence that oral splints reduce pain. Due to differences in splint type, the control group with no/minimal interventions and different types of TMD diagnoses between the individual studies, we were unable to investigate the heterogeneity any further. There were fewer studies and patients for the other time periods (>3 to 6 months: 2 trials, 160 patients and >6 to 12 months: 2 trials, three pairwise comparisons, 246 patients) and the effect sizes also failed to demonstrate that oral splints reduced pain.

**Table 1:** Splint versus no/minimal treatment in TMD patients, outcome: Pain: any combinable scale (higher = more pain) - 0 to 3 months

Study or Subgroup	Splint			Control			Weight	Std.Mean Difference IV, Random, 95% CI	Std.Mean Difference IV, Random, 95% CI
	Mean	SD	Total	Mean	SD	Total			
Conti 2015 (1)	9.23	22.5	12	37.62	22.5	9	3.8%	-1.21 [-2.17, -0.26]	**Figure:** 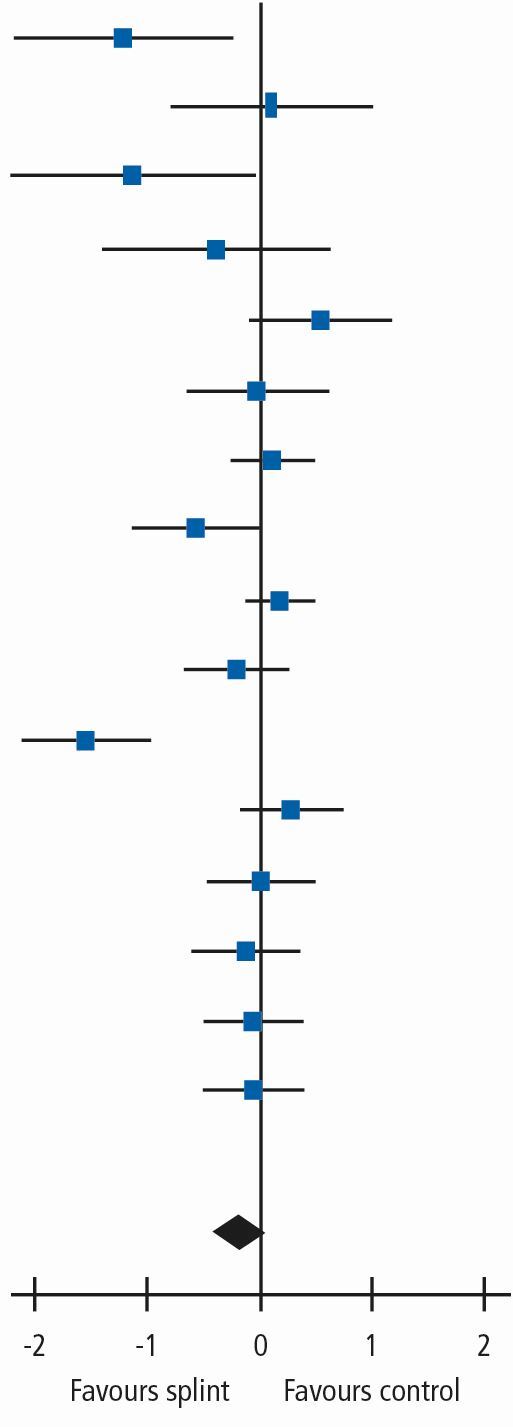
De Felicio 2010 (2)	14.56	8.14	10	13.6	9.49	10	4.2%	0.10 [-0.77, 0.98]	
Giannakopoulos 2016 (3)	16.7	17.8	12	40.8	25	6	3.3%	-1.13 [-2.19, -0.06]	
Giannakopoulos 2016 (4)	30	27	12	40.8	25	6	3.6%	-0.39 [-1.38, 0.60]	
Haketa 2010 (5)	36.5	28.7	25	21.3	26.4	19	6.0%	0.54 [-0.07, 1.15]	
Hasanoglu 2017 (1)	23	27.8	20	23.6	23.8	20	5.9%	-0.02 [-0.64, 0.60]	
Leeson 2007 (1)	41.1	26.2	62	38	28.1	63	8.1%	0.11 [-0.24, 0.46]	
List 1992 (6)	18	17	34	28	18	22	6.5%	-0.57 [-1.11, -0.02]	
Nagata 2015 (7)	11.571	19.8797	96	8.268	15.6888	85	8.5%	0.18 [-0.11, 0.47]	
Niemela 2012 (1)	34	32	39	40	26	37	7.2%	-0.20 [-0.65, 0.25]	
Nitecka-Buchta 2014 (1)	10	10.4	35	40	26	30	6.4%	-1.54 [-2.10, -0.98]	
Tatli 2017 (1)	20	19	40	15	17	40	7.3%	0.27 [-0.17, 0.72]	
Truelove 2006 (8)	47.83	20	56	47.6	20	27	7.2%	0.01 [-0.45, 0.47]	
Truelove 2006 (9)	45.08	20	54	47.6	20	27	7.1%	-0.12 [-0.59, 0.34]	
Yu 2016 (10)	20.5	8.7	42	21	11.7	42	7.4%	-0.05 [-0.48, 0.38]	
Yu 2016 (11)	19.7	9.3	42	20.2	8.9	42	7.4%	-0.05 [-0.48, 0.37]	
-									
**Total (95% CI)**			**591**			**485**	**100.0%**	**-0.18 [-0.42, 0.06]**	
Heterogeneity: Tau^2^ = 0.15; Chi^2^ = 50.22, df = 15 (P <0.0001); I^2^ = 70%									
Test for overall effect: Z = 1.50 (P = 0.13)									
Footnotes (1) Current pain intensity 0 to 100 mm VAS (2) Muscle pain 0 to 10 for: 1) when waking, 2) chewing, 3) speaking, 4) at rest; score summed = 0 to 40 scale (3) Current pain intensity 0 to 100 NRS (custom splint) (4) Current pain intensity 0 to 100 NRS (prefabricated splint) (5) Current maximum daily pain intensity 0 to 100 mm VAS (6) 0 to 100 mm VAS; recorded 3 times daily with average calculated on weekly basis (7) Current orofacial pain 0 to 100 NRS (8) CPI 0 to 100 - SD is median value from range of SDs reported in the paper (prefabricated splint) (9) CPI 0 to 100 - SD is median value from range of SDs reported in the paper (custom splint) (10) Current pain intensity 0 to 100 VAS (splint vs control) (11) Current pain intensity 0 to 100 VAS (splint + manipulative and physical therapies vs manipulative and physical therapies)									

There was no evidence of adverse events associated with splints, but reporting was poor regarding this outcome.

There was also no evidence that splints reduced TMD clicking or increased mouth opening, or improved quality of life, at any of the time points measured (online-only Supplementary Appendix 4). The certainty of the evidence for all these other outcomes was assessed as very low.

For TMD patients, we planned to undertake a sensitivity analysis restricted to trials where the inclusion criteria were based on, or could be clearly mapped to, one of the sets of diagnostic criteria mentioned in the Methods section. For the primary analysis of splints versus no/minimal intervention at 0 to 3 months (Table 1), there was no difference in the result when removing those trials that did not use the above diagnostic criteria: SMD -0.24 (95% CI -0.52 to 0.04; P = 0.09; I^2^ = 71%; 851 participants).

We carried out a sensitivity analysis restricting the meta-analysis in Table 1 to studies that measured pain at the time of assessment (current pain) measured on a 0 to 100 VAS or NRS. The results were consistent with the main SMD results: MD -4.48 (95% CI -11.59 to 2.64; P = 0.22; I^2^ = 94%; 874 participants).

We also carried out a sensitivity analysis restricting the meta-analysis in Table 1 to studies using stabilisation splints. Again, this did not change the result: SMD 0.04 (95% CI -0.13 to 0.22; P = 0.62; I^2^ = 27%; 750 participants). This removed much of the heterogeneity seen in the other analyses.

### Patients with bruxism

Only one of the studies focusing on patients with bruxism provided usable outcome data at 0 to 3 months;^45^ however, no studies looked at the primary outcome tooth wear. The aforementioned study on 78 patients looked at the other primary outcome pain on a 0 to 10 scale and indicated that splints reduced pain MD -2.01 (95% CI -2.62, -1.40).

## Discussion

### Summary of main results

Despite the inclusion of 35 studies comparing oral splints to no splints or a minimal intervention in patients with TMD, the body of evidence was assessed as being at very low certainty (see Summary of findings table in online-only Supplementary Appendix 5). There was no evidence that oral splints improved the following outcomes: pain; clicking of the temporomandibular joint; restricted mouth opening; or quality of life. 

For patients with bruxism, there was insufficient evidence to conclude whether the provision of oral splints reduced tooth wear, as no studies reported this. Although a small number of studies reported pain and other outcomes, there was also insufficient evidence to conclude whether or not oral splints were beneficial. We were unable to undertake any sensitivity analyses due to the lack of outcome data.

For the TMD patients, we undertook three separate sensitivity analyses restricted to trials where: a) the inclusion criteria were based on, or could be clearly mapped to, specific pre-determined sets of diagnostic criteria; b) only stabilisation splints were used; and c) current pain was measured on a 0 to 100 visual analogue scale or numerical rating scale. There were no differences in the results based on these factors.

For both patients with TMD and bruxism, due to differences in the diagnoses of the included trial participants and differences in the types of splints and control groups used, the applicability of the evidence is questionable and certainly incomplete for patients with bruxism.

Pain was reported in numerous different ways, at different times, and this reduced the number of studies that could be combined in a meta-analysis to produce a pooled estimate. The use of an agreed measure for pain and how and when this is measured would enable the pain data from all studies to contribute to a single pooled estimate. It is also important to consider what would be a clinically important reduction in pain. It is suggested that a reduction of around 20% represents a minimally important decrease, 30% a moderately important decrease and 50% a substantial decrease.^48^

Numerous studies reported on some of our outcomes but did not report the data in a suitable format for inclusion in our meta-analyses (including missing standard deviations). This can mean that meta-analyses are biased due to missing information. This highlights the need for standardisation in both 'what to measure' and 'how to measure it' in clinical trials within this area of research; otherwise, there will continue to be research waste, with data that we are unable to pool in data syntheses. Initiatives such as IMMPACT (Initiative on Methods, Measurement, and Pain Assessment in Clinical Trials), COMET (Core Outcome Measures in Effectiveness Trials) and COSMIN (COnsensus-based Standards for the selection of health Measurement INstruments) could help with these issues.

### Certainty of the evidence

The certainty of the evidence for comparing splints with no splints/minimal interventions in patients with all sub-types of TMD was downgraded to 'very low' due to the studies being of high risk of bias, heterogeneity and lack of precision in the estimates (see online-only Supplementary Appendix 5). Most studies were assessed as being at high risk of bias due to the inability of researchers to blind patients to wearing a splint or not. As the primary outcome for the TMD patients was pain assessed by the patients themselves, this meant that outcome measurement was also assessed as being at high risk of bias. This risk of bias does not necessarily reflect how well the studies have been conducted as it is not possible to design trials to overcome this problem. However, the bias should still be acknowledged when considering our overall certainty of the evidence. If blinding was to be disregarded, our certainty of the evidence would still remain low.

There were no studies looking at tooth wear, and very few studies and lack of useable other data for the patients with bruxism; therefore we were unable to determine whether splints were effective in these patients.

The risk of bias for all the studies was high. Although patient blinding is not possible when comparing oral splints with no splints or a minimal intervention, there were also problems with selective reporting bias and incomplete outcome data.

### Alternative treatment options for TMD

Other recent research into treatment options for the management of TMD has included orthognathic surgery,^49^ TMJ lavage,^50^ physiotherapy,^51^^,^^52^ low-level laser therapy,^53^^,^^54^ exercise therapy,^55^ pharmacological treatment, ^56^ and acupuncture. ^57^ However, results are mixed and generally unconvincing.

## Conclusions

### Implications for healthcare

From this systematic review, there is no clear evidence to support the provision of splints for the various sub-types of TMD or bruxism. However, the body of evidence that this conclusion is based on is of very low certainty. The studies included in this review differed in three important factors: 1) diagnoses, 2) splint type, and 3) outcome measurement/reporting. This made it difficult to draw clear and definitive conclusions.

### Recommendations for future research

Further well-conducted randomised controlled trials are urgently needed to determine whether the use of splints is clinically effective, generates meaningful patient benefit and whether splints offer an efficient use of resources in both Bruxism and TMD. Multiple trials will be required to concentrate on specific sub-types of TMD in order to facilitate future, more focused meta-analyses. The need for further trials is perhaps more pronounced in bruxism patients, as there were no trials measuring tooth wear.

## Electronic supplementary material

Supplementary Appendices 1-5 (PDF 842KB)
