# Supplementary material for: Oral splints for temporomandibular disorder or bruxism: a systematic review
Source: Br Dent J. 2020 Feb 14;228(3):191–7. doi: 10.1038/s41415-020-1250-2 (PMC7718146; doi:10.1038/s41415-020-1250-2)
Supplement: Supplementary file 1 — Supplementary Appendices 1-5 (PDF 842KB) [file 41415_2020_1250_MOESM1_ESM.pdf]

Supplementary Appendix 1: Electronic searches

Cochrane Central Register of Controlled Trials (CENTRAL) search strategy

#1 [mh ^"Occlusal adjustment"]
#2 [mh ^"Occlusal splints"]
#3 [mh ^"Orthodontic appliances"]
#4 ((occlusal or oral or temporomandibular or jaw* or mandib* or mouth* or bite* or TMJ or dental) near/5 splint*)
#5 ((dental or mouth or gum) next (guard* or shield*))
#6 (mouthguard* or gumguard* or nightguard* or gumshield* or "bite plane*" or toothprotector*
or "tooth protector*")
#7 "splint therapy"
#8 ((oral or TMJ or orofacial) next appliance*)
#9 {or #1-#8}
#10 [mh "craniomandibular disorders"]
#11 [mh ^"facial pain"]
#12 [mh ^"facial neuralgia"]
#13 [mh ^"trigeminal neuralgia"]
#14 [mh ^arthralgia]
#15 [mh ^"temporomandibular joint"]
#16 #14 and #15
#17 [mh bruxism]
#18 (bruxism or (teeth near/5 grind*) or (teeth near/5 clench) or (jaw* near/5 clench) or (jaw* near/5 grind*))
#19 ((craniofacial or myofacial or myofascial or facial or orofacial) near/5 (pain* or syndrome*))
#20 ("trigeminal neuralgia" or "sphenopalatine neuralgia" or "Costen* syndrome*")
#21 (("temporomandibular joint" or craniomandibular or jaw* or mandib*) near/5 (pain* or disorder* or dysfunction* or arthralgia or syndrome*))
#22 (TMD or TMJD or (TMJ near/3 (disorder* or dysfunction* or syndrome* or pain*))):ti,ab
#23 ((temporomandibular or jaw* or mandib*) near/5 (disk or disc) next displac*)
#24 #10 or #11 or #12 or #13 or #16 or #17 or #18 or #19 or #20 or #21 or #22 or #23
#25 #9 and #24

MEDLINE Ovid search strategy

1. Occlusal adjustment/
2. Occlusal splints/
3. Orthodontic appliances/
4. ((occlusal or oral or temporomandibular or jaw$ or mandib$ or mouth$ or bite$ or TMJ or dental) adj5 splint$).mp.
5. ((dental or mouth or gum) adj (guard$ or shield$)).mp.
6. (mouthguard$ or gumguard$ or nightguard$ or gumshield$ or "bite plane$" or toothprotector$ or "tooth protector$").mp.

7. "splint therapy".mp.
8. ((oral or TMJ or orofacial) adj appliance$).mp.
9. or/1-8
10. exp Craniomandibular disorders/
11. Facial pain/
12. Facial neuralgia/
13. Trigeminal neuralgia/
14. Arthralgia/ and temporomandibular joint/
15. exp bruxism/
16. (bruxism or (teeth adj5 grind$) or (teeth adj5 clench) or (jaw$ adj5 clench) or (jaw$ adj5 grind$)).mp.
17. ((craniofacial or myofacial or myofascial or facial or orofacial) adj5 (pain$ or syndrome$)).mp.
18. ("trigeminal neuralgia" or "sphenopalatine neuralgia" or "Costen$ syndrome$").mp.
19. (("temporomandibular joint" or craniomandibular or jaw$ or mandib$) adj5 (pain$ or disorder$ or dysfunction$ or arthralgia or syndrome$)).mp.
20. (TMD or TMJD or (TMJ adj3 (disorder$ or dysfunction$ or syndrome$ or pain$))).ti,ab.
21. ((temporomandibular or jaw$ or mandib$) adj5 (disk or disc) adj displac$).mp.
22. or/10-21
23. 9 and 22

Cochrane Search filter for MEDLINE Ovid

Cochrane Highly Sensitive Search Strategy (CHSSS) for identifying randomized trials in MEDLINE: sensitivity maximising version (2008 revision) as referenced in Chapter 6.4.11.1 and detailed in box 6.4.c of The Cochrane Handbook for Systematic Reviews of Interventions Version 5.1.0 [updated March 2011].

1. randomized controlled trial.pt.
2. controlled clinical trial.pt.
3. randomized.ab.
4. placebo.ab.
5. drug therapy.fs.
6. randomly.ab.
7. trial.ab.
8. groups.ab.
9. or/1-8
10. exp animals/ not humans.sh.
11. 9 not 10

Embase Ovid search strategy

1. Occlusal splint/
2. Orthodontic device/
3. ((occlusal or oral or temporomandibular or jaw$ or mandib$ or mouth$ or bite$ or TMJ or dental) adj5 splint$).mp.

4. ((dental or mouth or gum) adj (guard$ or shield$)).mp.
5. (mouthguard$ or gumguard$ or nightguard$ or gumshield$ or "bite plane$" or toothprotector$ or "tooth protector$").mp.
6. "splint therapy".mp.
7. ((oral or TMJ or orofacial) adj appliance$).mp.
8. or/1-7
9. Temporomandibular joint disorder/
10. Face pain/
11. Trigeminus neuralgia/
12. Arthralgia/ and temporomandibular joint/
13. exp bruxism/
14. (bruxism or (teeth adj5 grind$) or (teeth adj5 clench) or (jaw$ adj5 clench) or (jaw$ adj5 grind$)).mp.
15. ((craniofacial or myofacial or myofascial or facial or orofacial) adj5 (pain$ or syndrome$)).mp.
16. ("trigeminal neuralgia" or "sphenopalatine neuralgia" or "Costen$ syndrome$").mp.
17. (("temporomandibular joint" or craniomandibular or jaw$ or mandib$) adj5 (pain$ or disorder$ or dysfunction$ or arthralgia or syndrome$)).mp.
18. (TMD or TMJD or (TMJ adj3 (disorder$ or dysfunction$ or syndrome$ or pain$))).ti,ab.
19. ((temporomandibular or jaw$ or mandib$) adj5 (disk or disc) adj displac$).mp.
20. or/9-19
21. 8 and 20

The above subject search was linked to adapted version of the Cochrane Embase Project filter for identifying RCTs in Embase Ovid (see http://www.cochranelibrary.com/help/central-creation-details.html for information):

1. Randomized controlled trial/
2. Controlled clinical study/
3. Random$.ti,ab.
4. randomization/
5. intermethod comparison/
6. placebo.ti,ab.
7. (compare or compared or comparison).ti.
8. ((evaluated or evaluate or evaluating or assessed or assess) and (compare or compared or comparing or comparison)).ab.
9. (open adj label).ti,ab.
10. ((double or single or doubly or singly) adj (blind or blinded or blindly)).ti,ab.
11. double blind procedure/
12. parallel group$1.ti,ab.
13. (crossover or cross over).ti,ab.
14. ((assign$ or match or matched or allocation) adj5 (alternate or group$1 or intervention$1 or patient$1 or subject$1 or participant$1)).ti,ab.
15. (assigned or allocated).ti,ab.
16. (controlled adj7 (study or design or trial)).ti,ab.
17. (volunteer or volunteers).ti,ab.
18. trial.ti.
19. or/1-18

20. (exp animal/ or animal.hw. or nonhuman/) not (exp human/ or human cell/ or (human or humans).ti.)
21. 19 not 20

CINAHL EBSCO search strategy

S22 S8 and S21
S21 S9 or S10 or S11 or S12 or S13 or S14 or S15 or S16 or S17 or S18 or S19 or S20
S20 ((temporomandibular or jaw* or mandib*) N5 (disk or disc))
S19 (TMD or TMJD or (TMJ N3 (disorder* or dysfunction* or syndrome* or pain*)))
S18 (("temporomandibular joint" or craniomandibular or jaw* or mandib*) N5 (pain* or disorder* or dysfunction* or arthralgia or syndrome*))
S17 ("trigeminal neuralgia" or "sphenopalatine neuralgia" or "Costen* syndrome*")
S16 ((craniofacial or myofacial or myofascial or facial or orofacial) N5 (pain* or syndrome*))
S15 (bruxism or (teeth N5 grind*) or (teeth N5 clench) or (jaw* N5 clench) or (jaw* N5 grind*))
S14 (MH bruxism+)
S13 (MH arthralgia) AND (MH "temporomandibular joint")
S12 (MH "trigeminal neuralgia")
S11 (MH "facial neuralgia")
S10 (MH "facial pain")
S9 (MH "craniomandibular disorders+")
S8 S1 or S2 or S3 or S4 or S5 or S6 or S7
S7 ((oral or TMJ or orofacial) N1 appliance*)
S6 "splint therapy"
S5 ((dental or mouth or gum) N1 (mouthguard* or gumguard* or nightguard* or gumshield* or "bite plane*" or toothprotector* or "tooth protector*") guard* or shield*))
S4 ((dental or mouth or gum) N1 (guard* or shield*))
S3 ((occlusal or oral or temporomandibular or jaw* or mandib* or mouth* or bite* or TMJ or dental) N5 splint*)
S2 (MH "Orthodontic appliances")
S1 (MH "Splints")

The above subject search was linked to Cochrane Oral Health’s filter for identifying RCTs in CINAHL EBSCO:

S1 MH Random Assignment or MH Single-blind Studies or MH Double-blind Studies or MH Triple-blind Studies or MH Crossover design or MH Factorial Design
S2 TI ("multicentre study" or "multicenter study" or "multi-centre study" or "multi-center study") or AB ("multicentre study" or "multicenter study" or "multi-centre study" or "multi-center study") or SU ("multicentre study" or "multicenter study" or "multi-centre study" or "multi-center study")
S3 TI random* or AB random*
S4 AB "latin square" or TI "latin square"

S5 TI (crossover or cross-over) or AB (crossover or cross-over) or SU (crossover or cross-over)
S6 MH Placebos
S7 AB (singl* or doubl* or trebl* or tripl*) or TI (singl* or doubl* or trebl* or tripl*)
S8 TI blind* or AB mask* or AB blind* or TI mask*
S9 S7 and S8
S10 TI Placebo* or AB Placebo* or SU Placebo*
S11 MH Clinical Trials
S12 TI (Clinical AND Trial) or AB (Clinical AND Trial) or SU (Clinical AND Trial)
S13 S1 or S2 or S3 or S4 or S5 or S6 or S9 or S10 or S11 or S12

Proquest Dissertation and Theses search strategy

all((splint or guard or shield or mouthguard or gumguard or gumshield or mouthshield or "tooth protector" or orthodontic)) AND all(("temporomandibular joint" or TMD or TMJD or "facial pain" or (face and pain) or bruxism))

Web of Science Conference Proceedings search strategy

15 #6 and #14
14 #7 or #8 or #9 or #10 or #11 or #12 or #13
13 TS=((temporomandibular or jaw* or mandib*) AND (disk or disc))
12 TS=(TMJ AND (disorder* or dysfunction* or syndrome* or pain*))
11 TS=(TMD or TMJD)
10 TS=(("temporomandibular joint" or craniomandibular or jaw* or mandib*) AND (pain* or disorder* or dysfunction* or arthralgia or syndrome*))
9 TS=("trigeminal neuralgia" or "sphenopalatine neuralgia" or "Costen* syndrome*")
8 TS=((craniofacial or myofacial or myofascial or facial or orofacial) AND (pain* or syndrome*))
7 TS=(bruxism or (teeth and grind*) or (teeth and clench) or (jaw* and clench) or (jaw* and grind*))
6 #1 or #2 or #3 or #4 or #5
5 TS=((oral or TMJ or orofacial) AND appliance*)
4 TS="splint therapy"
3 TS=((dental or mouth or gum) and (guard* or shield*))
2 TS=(mouthguard* or gumguard* or nightguard* or gumshield* or "bite plane*" or toothprotector or “tooth protector*)
1 TS=((occlusal or oral or temporomandibular or jaw* or mandib* or mouth* or bite* or TMJ or dental) AND splint*)

US National Institutes of Health Trials Registry (ClinicalTrials.gov) search strategy

Condition: temporomandibular joint disorder
Other terms: splint*

Condition: Facial pain

Other terms: splint*

WHO International Clinical Trials Registry Platform search strategy

Condition: temporomandibular joint disorder
Intervention: splint*

Condition: face AND pain
Intervention: splint*

American Academy of Dental Sleep Medicine website search strategy

temporomandibular and splint

IADR conference abstracts search strategy

occlusal splint and temporomandibular
occlusal splint and pain
occlusal splint and bruxism

Supplementary Appendix 2: Characteristics and risk of bias of included studies

Conti 2005 [30]	
**Characteristics**	
Study details	**Trial design:** parallel (3 arms)<br>**Location:** Orofacial Pain Clinic at Bauru Dental School, University of São Paulo, Brazil<br>**Number of centres:** one<br>Recruitment period: not reported<br>**Sample size calculation:** not reported<br>**Funding:** public (CAPES - Coordenação de Aperfeiçoamento de Pessoal de Nível Superior - Brazilian Government)<br>**Declarations/conflicts of interest:** not reported<br>* We emailed authors for data but none provided so far
Participants	**Diagnosis:** presence of TMJ disc displacement with reduction and chief complaint of pain in the joint followed by positive TMJ tenderness to manual palpation, accompanied or not by muscle symptoms. The presence of at least a clicking joint during opening, eliminated on opening in protrusion was also an inclusion criterion<br>**Duration since presenting condition began:** not reported<br>**Age at baseline (years):** Gp A (stabilisation splint): mean 32.7; Gp B (repositioning splint): mean 31.4; Gp C (no treatment): mean 31.1<br>**Gender:** not reported<br>**Number randomised:** 60<br>**Number evaluated:** 52
Interventions	**Comparison:** splint vs no splint for TMD<br>**Gp A:**<br>• Splint type: custom stabilisation splint<br>• Upper/lower jaw: not reported<br>• Material: not reported<br>• Teeth coverage: unclear<br>• Details of impression taking: not reported<br>• Instructions to patients: wear at night and when sleeping

	• Monitoring of patients: only at planned visits (1, 2 weeks, 1, 3, 6, 12 months)<br>**Gp B:**<br>• Splint type: custom anterior repositioning splint for 3 to 4 months and then converted into stabilisation splints for the remainder of the treatment period<br>• Upper/lower jaw: not reported<br>• Material: not reported<br>• Teeth coverage: unclear<br>• Details of impression taking: not reported<br>• Instructions to patients: wear at night and when sleeping for repositioning splint (not reported for stabilisation splint)<br>• Monitoring of patients: only at planned visits (1, 2 weeks, 1, 3, 6, 12 months)<br>**Gp C:** no treatment or initial counselling<br>**Duration of treatment:** 12 months
Outcomes	Assessed at 1, 2 weeks, 1, 3, 6, 12 months: we would have used the the 3, 6 and 12 month data in our 0 to 3 month, > 3 to 6 month, and > 6 to 12 month analyses respectively<br>**Primary:**<br>• Pain:<br>1) pain on 0 to 100 VAS (higher = more pain) (no usable data - no SD/SE/P-values)<br>2) pain on TMJ and masticatory and cervical muscle palpation (digital pressure of 1.5 kg) (no usable data - no mean + SD/SE/P-values or incidence data)<br>**Secondary:**<br>• TMJ clicking: presence of joint noises (detected during TMJ palpation) no usable data - no mean + SD/SE/P-values)<br>• Change in restricted mouth opening: maximum mouth opening (mm) (no usable data - no mean + SD/SE/P-values)

Risk of bias

Random sequence generation (selection bias)	Unclear risk of bias	"subjects were randomly located into one of the following groups"<br>Comment: insufficient information

Allocation concealment (selection bias)	Unclear risk of bias	"subjects were randomly located into one of the following groups"<br>Comment: insufficient information
Blinding of participants and personnel (performance bias)	High risk of bias	Unable to blind patients
Blinding of outcome assessment (detection bias)	High risk of bias	Subjective outcomes assessment by patients (except for 'TMJ clicking' and 'change in restricted mouth opening' which may be considered objective and were measured by a blinded assessor)
Incomplete outcome data (attrition bias)	Unclear risk of bias	Numbers per group at randomisation and assessment points were not reported
Selective reporting (reporting bias)	High risk of bias	Results very poorly reported with very limited data for all outcomes
Other bias	Unclear risk of bias	Level of reporting extremely poor so unable to assess this

**Conti 2012** [11]	
**Characteristics**	
Study details	**Trial design:** parallel (3 arms)<br>**Location:** Bauru School of Dentistry, University of Sao Paulo, Brazil<br>**Number of centres:** 1<br>**Recruitment period:** Not reported<br>**Sample size calculation:** not reported<br>**Funding:** public (supported by Conselho Nacional de Desenvolvimento Científico e Tecnológico (CNPq), Brazil)<br>**Declarations/conflicts of interest:** "The authors declare that they have no conflicts of interest"<br>* We emailed authors for data but none provided so far

Participants	**Diagnosis:** RDC/TMD - myofascial pain with or without jaw opening limitation (Ia and Ib)<br>**Duration since presenting condition began:** not reported<br>**Age at baseline (years):** Gp A: mean 38.1; Gp B: mean 35.3; Gp C: mean 38.1<br>**Gender:** Gp A: 19% male; Gp B: 12% male; Gp C: 0% male<br>**Number randomised:** 51 (Gp A: 21; Gp B: 16; Gp C: 14)<br>**Number evaluated:** at 3 months = 39 (Gp A: 17; Gp B: 13; Gp C: 9)
Interventions	**Comparison:** splint vs no splint for TMD<br>All patients received counselling for habits and behavioural changes (reinforced at each visit): instructed about beneficial behavioural changes and received a printed version of the instructions, containing information about relaxation techniques, sleep hygiene, diet modification, thermotherapy and massage in the painful area, as well as avoidance of caffeine and daytime clenching<br>**Gp A:**<br>• Splint type: custom occlusal stabilisation splint<br>• Upper jaw<br>• Material: hard (acrylic)<br>• Teeth coverage: full<br>• Details of impression taking: not reported<br>• Instructions to patients: advised to wear the appliance only at night while sleeping<br>• Monitoring of patients: seen at 2, 6 weeks and 3 months for adjustments<br>**Gp B:**<br>• Splint type: custom occlusal nociceptive trigeminal inhibition (NTI) splint<br>• Upper jaw<br>• Material: not reported<br>• Teeth coverage: partial<br>• Details of impression taking: not reported<br>• Instructions to patients: as above<br>• Monitoring of patients: as above<br>**Gp C:** no other treatment<br>**Duration of treatment:** 3 months
Outcomes	Assessed at 2, 6 weeks, 3 months: we used the 3 month data for our 0 to 3 month analysis

	**Primary:**<br>• Pain:<br>1) current pain 0 (no pain) to 100 (worst pain) mm VAS (no usable data - no SD/SE/P-values)<br>2) pressure pain threshold (PPT): digital algometer used to put pressure on muscles (patient presses button when feel pain); reported as kgf/cm^2 (higher score = less pain) (reported separately for left and right side for 5 muscles - data not used)<br>3) incidence of patients who halved their VAS scores	
**Risk of bias**		
Random sequence generation (selection bias)	Unclear risk of bias	"the patients were randomly allocated into one of the following three groups"<br>Comment: insufficient information
Allocation concealment (selection bias)	Unclear risk of bias	"the patients were randomly allocated into one of the following three groups"<br>Comment: insufficient information
Blinding of participants and personnel (performance bias)	High risk of bias	Unable to blind patients
Blinding of outcome assessment (detection bias)	High risk of bias	Subjective outcomes assessment by patients
Incomplete outcome data (attrition bias)	High risk of bias	Very high overall attrition 24% and especially high in the control group (Gp A: 19%; Gp B: 19%; Gp C: 36%)
Selective reporting (reporting bias)	High risk of bias	Data not adequately reported for pain on 0 to 100 VAS

Other bias	Unclear risk of bias	Lacking in detail so unable to assess

**Conti 2015** [12]	
**Characteristics**	
Study details	**Trial design:** parallel (3 arms)<br>**Location:** Orofacial Pain Clinic at Bauru Dental School, University of São Paulo, Brazil<br>**Number of centres:** 1<br>**Recruitment period**: not reported<br>**Sample size calculation:** not reported<br>**Funding**: not reported<br>**Declarations/conflicts of interest**: not reported<br>* We emailed authors for data but none provided so far
Participants	**Diagnosis:** disc displacement with reduction (**II**a) and arthralgia (**III**a) according to RDC-TMD (myofascial pain, disc displacement without reduction and osteoarthritis according to RDC-TMD were all excluded)<br>**Duration since presenting condition began:** not reported<br>**Age at baseline (years):** Gp A: mean 38.4; Gp B: mean 38.4; Gp C: mean 46<br>**Gender:** 3% male (not reported by group)<br>**Number randomised:** 60 (Gp A: 20; Gp B: 20; Gp C: 20)<br>**Number evaluated:** 3 months: 33 (Gp A: 12; Gp B: 12; Gp C: 9
Interventions	**Comparison:** splint vs no splint for TMD<br>All patients received counselling: instructions containing information about relaxation techniques, sleep hygiene, diet modification, hot thermotherapy, as well as avoidance of caffeine and awaking clenching<br>**Gp A:**<br>• Splint type: custom anterior repositioning occlusal splint<br>• Upper jaw<br>• Material: hard (acrylic)<br>• Teeth coverage: unclear<br>• Details of impression taking: not reported<br>• Instructions to patients: wear only while sleeping

	• Monitoring of patients: visits at 2 weeks, 6 weeks and 3 months: in each visit, a comprehensive assessment of splint adjustments was performed for and the counseling and behavioral changes information were reinforced in all groups<br>**Gp B:**<br>• Splint type: custom Nociceptive Trigeminal Inhibition Clenching Suppression System (NTI-tss) occlusal splint<br>• Upper jaw<br>• Material: not reported<br>• Teeth coverage: partial<br>• Details of impression taking: not reported<br>• Instructions to patients: as above<br>• Monitoring of patients: as above<br>**Gp C:** no other treatment<br>**Duration of treatment:** 3 months
Outcomes	Assessed at 2, 6 weeks, 3 months: we used the 3 month data for our 0 to 3 month analysis<br>**Primary:**<br>• Pain:<br>1) current pain intensity 0 (no pain) to 100 (worst pain) mm VAS (reported by graph but no SDs - a P-value was presented for the comparison of Gp A vs Gp C so we have used this in the meta-analysis)<br>2) pressure pain threshold (PPT) of each TMJ, using a digital pressure algometer, where patients press button when they feel pain, reported at 3 months (data presented at 3 months as means and SDs for each joint, described as VAS score - not used)<br>**Secondary:**<br>• TMJ clicking: presence of TMJ sounds according to RDC/TMD. Data presented as bar charts with % joints on y axis, so not used due to clustering of data<br>• Change in restricted mouth opening: unassisted maximum mouth opening in mm (between the top and bottom edges, taking the midline as reference) until pain felt<br>• Patient satisfaction: comfort level reported at 2 weeks (more comfortable or not) - data reported only for splint groups so not usable in meta-analyses
**Risk of bias**	

Random sequence generation (selection bias)	Unclear risk of bias	"randomly assigned"<br>Comment: insufficient information
Allocation concealment (selection bias)	Unclear risk of bias	"randomly assigned"<br>Comment: insufficient information
Blinding of participants and personnel (performance bias)	High risk of bias	Unable to blind patients
Blinding of outcome assessment (detection bias)	High risk of bias	Subjective outcomes assessment by patients (except for 'TMJ clicking' and 'change in restricted mouth opening' which may be considered objective and were measured by a blinded assessor)
Incomplete outcome data (attrition bias)	High risk of bias	Overall attrition 32% at 6 weeks and 45% at 3 months
Selective reporting (reporting bias)	High risk of bias	Data not adequately reported (e.g. for VAS pain, no SD reported and P-value only reported for comparison between Gp A and Gp C)
Other bias	Unclear risk of bias	Lacking in detail so unable to assess

**Costa 2015** [13]	
**Characteristics**	
Study details	**Trial design:** parallel (2 arms)<br>**Location:** Orofacial Pain Clinic at Bauru Dental School, University of São Paulo, Brazil<br>**Number of centres:** 1<br>**Recruitment period:** August 2011 to November 2012<br>**Sample size calculation:** reported incompletely (unclear if met)

	**Funding:** public (grant no 2011/04441-6 from FAPESP - Sao Paulo Research Foundation)<br>**Declarations/conflicts of interest:** "The authors declare no conflicts of interest"<br>* We emailed authors for data but none provided so far
Participants	**Diagnosis:** RDC/TMD - myofascial pain<br>**Duration since presenting condition began:** pain duration at least 3 months<br>**Age at baseline (years):** (inclusion was 18 to 50) Gp A: mean 27.7 (SD 6.7); Gp B: mean 36 (SD 6.6)<br>**Gender:** Gp A: 10% male; Gp B: 10% male<br>**Number randomised:** 60 (Gp A: 30; Gp B: 30)<br>**Number evaluated:** 5 months: 41 (Gp A: 24; Gp B: 17); unclear how many participants were evaluated at 2 months
Interventions	**Comparison:** splint vs no splint for TMD<br>All patients received counselling: verbal and written information about TMD aetiology and prognostics, diet modification in the sense of avoiding hard foods, use of reminders to avoid parafunctional habits, relaxation techniques of the jaw, application of a heating pad on painful muscles, followed by stretching and self-massage, as well as sleep hygiene and encouragement to practice social and aerobic activities<br>**Gp A:**<br>• Splint type: custom occlusal stabilisation splint<br>• Upper jaw<br>• Material: hard (acrylic)<br>• Teeth coverage: full<br>• Details of impression taking: not reported<br>• Instructions to patients: wear splints at night only while sleeping<br>• Monitoring of patients: adjustments during visits at 2 and 5 months<br>**Gp B:** no other treatment<br>**Duration of treatment:** 5 months

Outcomes	Assessed at 2 and 5 months: we used the 5 month data for our > 3 to 6 month analysis (we were unable to use the 2 month data as the numbers analysed were not reported)<br>**Primary:**<br>• Pain: Catastrophizing Thoughts subscale of the Pain Related Self-Statement Scale. Self-reported questionnaire consisting of 9 statements related to catastrophizing thoughts involved in pain perception. Respondent asked to answer each statement indicating the frequency of thinking about pain during a pain crisis, on a 0 to 4 scale. The sum of all frequencies was divided by the total number of questions. Higher values demonstrate higher levels of pain catastrophizing (reported in additional table - not used for SMD of pain)<br>**Secondary:**<br>• Frequency of headaches (secondary to pain-related TMD): categorised as number having either infrequent/absent headache (< 1 day/month), frequent headache (1 to 14 days/month), or chronic headache (> 14 days/month) - we dichotomised the data as incidence of frequent or chronic headache<br>• Quality of life (including physical and emotional function): anxiety and depression reported using Hospital Anxiety and Depression scale (HADs). Self-reported questionnaire consisting of 14 multiple-choice questions involving two interspersed subscales, one for anxiety (7 questions) and the other for depression (7 questions). The scores ranged from 0 to 21 points and were divided into four categories: 0 to 7 (no anxiety or depression), 8 to 10 (mild anxiety or depression), 11 to 14 (moderate anxiety or depression) and 15 to 21 (severe anxiety or depression) - we dichotomised the data as incidence of moderate or severe anxiety/depression (data not used - some do not appear to add up to 100%)

Risk of bias

Random sequence generation (selection bias)	Low risk of bias	"randomly assigned, by a computer-generated list"<br>Comment: appropriate method
Allocation concealment (selection bias)	Low risk of bias	"The allocation of groups was concealed and designated according to sequentially numbered, opaque, sealed envelopes given to a person who did not know the allocation sequence"

		Comment: the next assignment was adequately concealed from the person randomising patients
Blinding of participants and personnel (performance bias)	High risk of bias	Unable to blind patients
Blinding of outcome assessment (detection bias)	High risk of bias	Subjective outcomes assessment by patients
Incomplete outcome data (attrition bias)	High risk of bias	Overall attrition at 5 months was 32% and also differed by group (Gp A: 20%; Gp B: 43%). This could potentially bias the results
Selective reporting (reporting bias)	High risk of bias	We would have expected the authors to also report a more simple pain intensity outcome in line with other RCTs in this field (e.g. 0 to 100 mm VAS)
Other bias	Low risk of bias	No apparent other bias

Daif 2012 [33]

**Characteristics**	
Study details	**Trial design:** parallel (2 arms)<br>**Location:** Department of Oral and Maxillofacial, Faculty of Oral & Dental Medicine, Cairo University, Egypt<br>**Number of centres:** 1<br>**Recruitment period:** not reported<br>**Sample size calculation:** not reported<br>**Funding:** not reported<br>**Declarations/conflicts of interest:** "The authors report no conflicts of interest"
Participants	**Diagnosis:** TMD with myofascial pain by the presence of a non teeth-related chronic orofacial pain with localized areas of tenderness in the

<table>
<tr><td colspan="2"></td><td>masticatory muscles. Signs and symptoms were recorded according to the clinical dysfunction index of Helkimo
Duration since presenting condition began: not reported
Age at baseline (years): overall: mean 32 years (range 22 to 46 years)
Gender: overall: 42.5% male
Number randomised: 40 (Gp A: 20; Gp B: 20)
Number evaluated: 40 (Gp A: 20; Gp B: 20)</td></tr>
<tr><td colspan="2">Interventions</td><td>**Comparison:** splint vs no splint for TMD
Gp A:
• Splint type: custom made flat-plane occlusal splint
• Upper jaw
• Material: hard (acrylic resin)
• Teeth coverage: full
• Details of impression taking: fabricated on articulated dental casts. The vertical pin of the articulator was adjusted to create a 2 to 3 mm space between the molars
• Instructions to patients: wear the splints during the whole night and as much as possible during the daytime for 6 months
• Monitoring of patients: not reported
Gp B: no treatment
Duration of treatment: 6 months</td></tr>
<tr><td colspan="2">Outcomes</td><td>Assessed at 6 months: grouped under > 3 to 6 months analysis
Secondary:
• Adherence to treatment: incidence of those not totally complying with postoperative instructions
The other outcome assessed at 6 months (Clinical Dysfunction Index of Helkimo) was not an outcome of this review</td></tr>
<tr><td colspan="3">**Risk of bias**</td></tr>
<tr><td>Random sequence generation (selection bias)</td><td>Low risk of bias</td><td>"randomization was performed using a computer-generated random number list"
Comment: appropriate method</td></tr>
<tr><td>Allocation concealment (selection bias)</td><td>Unclear risk of bias</td><td>"randomization was performed using a computer-generated random number list"
Comment: insufficient information</td></tr>
</table>

Blinding of participants and personnel (performance bias)	High risk of bias	Blinding not possible
Blinding of outcome assessment (detection bias)	High risk of bias	Patients were not blinded but self reported compliance
Incomplete outcome data (attrition bias)	Low risk of bias	All randomised participants were included in analysis
Selective reporting (reporting bias)	High risk of bias	The study focused on TMD with pain and therefore we would have expected pain to have been measured separately
Other bias	Low risk of bias	No apparent other bias

de Felicio 2006 [31]

Characteristics

Study details	**Trial design:** parallel (2 arms)<br>**Location:** Dental School of Ribeirão Preto of the University of São Paulo, Brazil<br>**Number of centres:** 1<br>**Recruitment period**: not reported<br>**Sample size calculation:** not reported<br>**Funding:** not reported<br>**Declarations/conflicts of interest:** not reported
Participants	**Diagnosis:** presence of signs and symptoms characteristic of TMD: pain in the masticatory muscles and/or in the TMJ during mandibular function and palpation of the structures, limitation or deviation of mandibular movements, noises in the TMJ, and abnormal static or dynamic occlusal relation

	**Duration since presenting condition began:** not reported<br>**Age at baseline (years):** not reported<br>**Gender:** not reported<br>**Number randomised:** 84 (Gp A: 42; Gp B: 42)<br>**Number evaluated:** 84 (Gp A: 42; Gp B: 42)
Interventions	**Comparison:** splint vs minimal treatment for TMD<br>**Gp A:**<br>• Splint type: custom occlusal splint<br>• Upper jaw<br>• Material: hard (heat-polymerizable colorless acrylic resin)<br>• Teeth coverage: full<br>• Details of impression taking: dental arches molded with irreversible hydrocolloid (alginate) and the plaster casts obtained were mounted on a semi-adjustable articulator in the mandibular position of centric relation<br>• Instructions to patients: use during the day and at night for the first 15 days, and only at night thereafter<br>• Monitoring of patients: not reported<br>**Gp B:** continued to attend occlusion outpatient clinic, receiving information about TMD<br>**Duration of treatment:** 50 days
Outcomes	Assessed at 50 days: grouped under 0 to 3 months analysis<br>**Primary:**<br>• Pain:<br>a) presence of muscular pain (yes/no)<br>b) severity of muscular pain and TMJ pain assessed separately using a 0 to 10 NRS - when waking up, during mastication, when speaking, and at rest all assessed separately for each type of pain and summed (no usable data - no mean with SD/SE/CI or P-value)<br>**Secondary:**<br>• TMJ clicking:<br>a) articular noise (yes/no) - "The predominant type of articular noise was a click (83.33% of cases)"<br>b) joint noise assessed using a 0 to 10 NRS - when waking up, during mastication, when speaking, and at rest all assessed separately and summed (no usable data - no mean with SD/SE/CI or P-value)<br>• Change in restricted mouth opening: difficulty opening mouth (yes/no)

Risk of bias		
Random sequence generation (selection bias)	Low risk of bias	"patients with TMD were randomly divided into two groups using GraphPad software"<br>Comment: author provided this information by email
Allocation concealment (selection bias)	Unclear risk of bias	Not mentioned
Blinding of participants and personnel (performance bias)	High risk of bias	Blinding not possible
Blinding of outcome assessment (detection bias)	High risk of bias	Subjective outcomes assessed by the patients
Incomplete outcome data (attrition bias)	Low risk of bias	All randomised patients were included in the analyses
Selective reporting (reporting bias)	High risk of bias	Poor reporting of NRS severity scores
Other bias	Low risk of bias	No apparent other bias

de Felicio 2010 [14]

Characteristics	
Study details	**Trial design:** parallel (4 arms)<br>**Location:** Faculty of Medicine of Ribeirão Preto of the University of São Paulo, Brazil<br>**Number of centres:** 1

	**Recruitment period:** not reported<br>**Sample size calculation:** not reported<br>**Funding:** public (supported by Fundação de Amparo à Pesquisa do Estado de São Paulo -FAPESP, Process N. 2004/08478-8 and Conselho Nacional de Pesquisa - CNPq, Process N. 300950/2007-1)<br>**Declarations/conflicts of interest:** not reported
Participants	**Diagnosis:** long-lasting associated articular and muscular TMD based on RDC/TMD<br>**Duration since presenting condition began:** mean duration of TMD was 74.4 months (range: 6 to 300 months)<br>**Age at baseline (years):** Gp A: mean 29 (range 17 to 64); Gp B: mean 34 (range 14 to 63)<br>**Gender:** not reported<br>**Number randomised:** 20 (Gp A: 10; Gp B: 10)<br>**Number evaluated:** 20 (Gp A: 10; Gp B: 10) - this is assumed as attrition was not mentioned
Interventions	**Comparison:** splint vs no splint for TMD<br>**Gp A:**<br>• Splint type: custom occlusal splint (Michigan)<br>• Upper/lower jaw not specified<br>• Material: not reported<br>• Teeth coverage: full<br>• Details of impression taking: not reported<br>• Instructions to patients: continuous use recommended during first 15 days, except during eating and teeth cleaning, followed by only night-time use after this period<br>• Monitoring of patients: not reported<br>**Gp B:** no treatment<br>**Gp C:** orofacial myofunctional therapy (not eligible for inclusion in this review)<br>**Gp D:** asymptomatic controls (not eligible for inclusion in this review)<br>**Duration of treatment:** 45 days
Outcomes	Assessed at 45 days: grouped under 0 to 3 months analysis<br>**Primary:**<br>• Pain: muscle pain assessed on a printed 0 (absence of symptom) to 10 (worst severity) for the following 4 situations: 1) when waking up, 2) during chewing, 3) when speaking, 4) at

	rest. The score was then summed and is therefore a 0 to 40 scale<br>**Secondary:**<br>• TMJ clicking: assessed on a printed 0 (absence of symptom) to 10 (worst severity) for the following 4 situations: 1) when waking up, 2) during chewing, 3) when speaking, 4) at rest. The score was then summed and is therefore a 0 to 40 scale<br>• Change in restricted mouth opening: maximal mandibular opening in mm (unclear if with/without/until pain or assisted/unassisted)

Risk of bias

Random sequence generation (selection bias)	Low risk of bias	"randomly assigned to three groups using the GraphPad software"<br>Comment: appropriate method
Allocation concealment (selection bias)	Unclear risk of bias	"randomly assigned to three groups using the GraphPad software"<br>Comment: insufficient information
Blinding of participants and personnel (performance bias)	High risk of bias	Blinding not possible
Blinding of outcome assessment (detection bias)	High risk of bias	Subjective outcomes assessment by patients (except for 'change in restricted mouth opening' and 'TMJ clicking' which were objective but blinded assessor not mentioned)
Incomplete outcome data (attrition bias)	Unclear risk of bias	Assuming no attrition but not entirely clear
Selective reporting (reporting bias)	Low risk of bias	Outcomes fully reported
Other bias	Low risk of bias	No other apparent bias

DeVocht 2013 [15]	
**Characteristics**	
Study details	**Trial design:** parallel (4 arms)<br>**Location:** Craniofacial Clinical Research Centre, University of Iowa, USA; Palmer College of Chiropractic, Davenport, Iowa, USA<br>**Number of centres:** 2<br>**Recruitment period:** over 18 months ending in July 2011<br>**Sample size calculation:** No ("We chose the sample size to determine feasibility and, therefore, the study was not powered to detect differences between groups)<br>**Funding:** public and industry (grants from National Institutes of Health; ineligible interventions mentioned above were provided by the manufacturer)<br>**Declarations/conflicts of interest:** one author declared instructing for Activator Methods International, Phoenix (manufacturers of the ineligible interventions mentioned above). None of the other authors reported any disclosures
Participants	**Diagnosis:** myofascial pain (RDC/TMD Axis I) with TMD pain over the previous week of at least a 3 on a 0 to 10 NRS<br>**Duration since presenting condition began:** (inclusion criteria required participants having had TMD symptoms for at least 6 months): Gp A: median 10 years (IQR 12.5); Gp B: median 10 years (IQR 11)<br>**Age at baseline (years):** Gp A: mean 31 (range 13 to 76); Gp B: mean 30 (range 15 to 72)<br>**Gender:** Gp A: 15% male; Gp B: 24% male<br>**Number randomised:** 41 (Gp A: 20; Gp B: 21)<br>**Number evaluated:** 41 (Gp A: 20; Gp B: 21) - ITT used (multiple imputation for the missing outcomes)
Interventions	**Comparison:** Splint vs no splint for TMD<br>All patients received TMD self-care program: similar to usual recommendations given to patients with TMD. Conservative and reversible strategies requiring the dentist or dental care co-ordinator to review TMD with the participant; explain to them the current

	understanding of prognosis; and provide standardised treatment checklist identifying recommendations for care (e.g. jaw relaxation, reduction of parafunctional behaviours, use of thermal packs, use of over-the-counter pain medications, passive jaw-opening stretches and suggestions about stress reduction)<br>**Gp A:**<br>• Splint type: custom reversible interocclusal splint therapy (RIST)<br>• Upper jaw<br>• Material: hard (acrylic)<br>• Teeth coverage: full<br>• Details of impression taking: maxillary and mandibular vinyl polysiloxane impressions made, then interocclusal records were made using a fast-setting vinyl polysiloxane bite registration material and an intraoral metal tray<br>• Instructions to patients: wear at night and for at least 2 hours during the day<br>• Monitoring of patients: none<br>**Gp B:** no other treatment<br>**Gp C:** Activator Method Chiropractic Technique (not eligible for inclusion in this review)<br>**Gp D:** sham Activator Method Chiropractic Technique (not eligible for inclusion in this review)<br>**Duration of treatment:** 2 months
Outcomes	Assessed at 2 and 6 months: we used these in our 0 to 3 month and > 3 to 6 month analyses respectively<br>**Primary:**<br>• Pain: current pain intensity using a 0 (no pain) to 10 (worst pain) NRS; reported as change score (unable to combine change score in primary M-A using SMD; used in sensitivity analyses of studies reporting current pain intensity on VAS/NRS at 0 to 3 months and > 3 to 6 months)<br>**Secondary:**<br>• Quality of life (including physical and emotional function): 14-item Oral Health Impact Profile (OHIP-14) - contains 2 questions about each of 7 dimensions, indicating how often the participant had experienced each difficulty in the previous month; possible responses range from 0 (never) to 4 (very often). The OHIP score is obtained by summing the 14 ratings; reported as change score (unable to combine change score in primary M-A using SMD; used in sensitivity analyses of studies reporting current pain intensity on VAS/NRS at 0 to 3 months)

	• Patient satisfaction: using a 0 (not at all satisfied) to 10 (extremely satisfied) NRS (no usable data at 6 months: no SD/SE/CI or P-value)

Risk of bias

Random sequence generation (selection bias)	Low risk of bias	"We allocated participants via a randomization algorithm stored in the Web-based system, with future allocations concealed"<br>Comment: appropriate method
Allocation concealment (selection bias)	Low risk of bias	"We allocated participants via a randomization algorithm stored in the Web-based system, with future allocations concealed"<br>Comment: probably done as a separate data co-ordinating centre was used (The office of Data Management and Biostatistics at the Palmer Centre for Chiropractic Research)
Blinding of participants and personnel (performance bias)	High risk of bias	Blinding not possible
Blinding of outcome assessment (detection bias)	High risk of bias	Subjective outcomes assessed by the patients
Incomplete outcome data (attrition bias)	Low risk of bias	ITT used (multiple imputation for the missing outcomes)
Selective reporting (reporting bias)	Low risk of bias	No evidence of selective reporting
Other bias	Low risk of bias	No other apparent bias

Elsharkawy 1995 [39]

Characteristics	
Study details	**Trial design:** parallel (4 arms)<br>**Location:** Oral Surgery Department, Cairo University, Egypt<br>**Number of centres:** 1<br>**Recruitment period:** not reported<br>Sample size calculation: not reported<br>**Funding:** not reported<br>**Declarations/conflicts of interest**: not reported
Participants	**Diagnosis:** based on presence of two or more of: TMJ pain and tenderness when palpated both laterally in the preauricular area and via the external auditory meatus, masticatory muscle tenderness, clicking and jaw locking, and trismus (patients with disc displacement were excluded)<br>**Duration since presenting condition began:** not reported<br>**Age at baseline (years):** not reported<br>**Gender:** not reported<br>**Number randomised:** 50 (Gp A: 25; Gp B: 25)<br>**Number evaluated:** 46 (Gp A: 23; Gp B: 23)
Interventions	**Comparison:** splint vs no splint for TMD<br>All patients in Gp A and Gp B received acuhealth therapy: acuhealth unit detects energy acupucture points and performs stimulation/treatment without penetrating the skin; weekly sessions for 8 weeks<br>**Gp A:**<br>• Splint type: custom occlusal splint<br>• Lower jaw<br>• Material: soft (polyvinyl)<br>• Teeth coverage: full<br>• Details of impression taking: not reported<br>• Instructions to patients: wear at night<br>• Monitoring of patients: not reported<br>**Gp B:** no other treatment<br>**Gp C*:** above mentioned splint-alone (no acuhealth therapy)

	**Gp D*:** placebo acuhealth therapy (machine switched off)<br>* Groups C and D are excluded from this review as it was not possible to make any eligible pairwise comparisons using them<br>**Duration of treatment:** 8 weeks	
Outcomes	Assessed at 3 months: grouped under 0 to 3 months analysis (also assessed at 6 and 12 months but patients had crossed over and were no longer analysed according to the group they were originally randomised to, so the data was not eligible for inclusion)<br>**Primary:**<br>• Pain:<br>a) current pain intensity 0 (no pain) to 100 (worst pain) mm VAS (no data reported)<br>b) subjective dysfunction score: 1 = no pain, 2 = mild pain, 3 = moderate pain, 4 = severe pain, 5 = very severe pain (no data reported)<br>c) the results for pain outcomes a and b above were individually assessed according to the following scale: impaired, unchanged, improved, symptom free (we dichotomised this as incidence of improved and symptom free)	
**Risk of bias**		
Random sequence generation (selection bias)	Unclear risk of bias	"randomly divided"<br>Comment: insufficient information
Allocation concealment (selection bias)	Unclear risk of bias	"randomly divided"<br>Comment: insufficient information
Blinding of participants and personnel (performance bias)	High risk of bias	Blinding not possible

Blinding of outcome assessment (detection bias)	High risk of bias	Subjective pain outcomes assessment by patients
Incomplete outcome data (attrition bias)	High risk of bias	We were unable to use data at 6 and 12 months as some patients were no longer analysed according to the group they were originally randomised to
Selective reporting (reporting bias)	High risk of bias	Incomplete reporting of pain data
Other bias	Low risk of bias	No other apparent bias

**Ficnar 2013** [16]	
**Characteristics**	
Study details	**Trial design:** parallel (3 arms)<br>**Location:** Department of Prosthetic Dentistry and Biomaterials and the Department of Orthodontics of the Center for Dental, Oral and Maxillofacial Diseases of Münster University Hospital, Germany<br>**Number of centres:** 1<br>**Recruitment period:** 2009 to 2010<br><br>**Sample size calculation:** not reported<br><br>**Funding:** industry ("The expenses for this study were payed by Jaxeurope")<br><br>**Declarations/conflicts of interest:** "The authors declare that they have no competing interests"
Participants	**Diagnosis:** RDC/TMD Ia or Ib (myofascial pain) also in combination with arthralgia (IIIa) and/or disk displacement with reduction (IIa) and a maximum "von Korff" pain grade of I (functional pain with low levels of intensity) to II (functional pain with high levels of intensity)<br>**Duration since presenting condition began:** not reported

	**Age at baseline (years):** median 35 (not reported by group)<br>**Gender:** 21% male (not reported by group)<br>**Number randomised:** 63 (Gp A: 21; Gp B: 21; Gp C: 21)<br>**Number evaluated:** 58 (Gp A: 18; Gp B: 21; Gp C: 19)
Interventions	**Comparison:**<br>1) splint vs no splint for TMD<br>2) prefabricated splint vs custom-made splint for TMD<br>All patients received conservative therapy: self-exercises (muscle exercise form according to Prof. Schulte, self-massage techniques, mouth opening exercises), medication-based therapy using NSAID, muscle relaxants as well as manual therapy<br>**Gp A:**<br>• Splint type: custom occlusal stabilisation splint<br>• Upper jaw/lower jaw: not reported<br>• Material: not reported<br>• Teeth coverage: full<br>• Details of impression taking: a bite registration was taken using Beauty Pink wax as registration plate and Aluwax<br>• Instructions to patients: wear every night and for 2 hours during the day<br>• Monitoring of patients: not reported<br>**Gp B:**<br>• Splint type: prefabricated, semi-finished occlusal splint (SOLUBrux)<br>• Upper jaw<br>• Material: soft (malleable thermoplastic)<br>• Teeth coverage: full<br>• Details of impression taking: no impression needed<br>• Instructions to patients: as above<br>• Monitoring of patients: not reported<br>**Gp C**: no other treatment<br>Duration of treatment: 2.5 months
Outcomes	Assessed at 2 weeks and 2.5 months: we would have used the 2.5 month data for our 0 to 3 month analysis<br>**Primary:**<br>• Pain: reduction in the number of of pressure-sensitive areas upon palpation of: a) masticatory muscles, b) TMJ (no usable data - medians presented)<br>**Secondary:**

	• Change in restricted mouth opening: unassisted pain free maximum jaw opening - incisal edge distance in mm (no usable data - medians presented)	
**Risk of bias**		
Random sequence generation (selection bias)	Unclear risk of bias	"randomisation"<br>Comment: insufficient information
Allocation concealment (selection bias)	Unclear risk of bias	"randomisation"<br>Comment: insufficient information
Blinding of participants and personnel (performance bias)	High risk of bias	Blinding not possible
Blinding of outcome assessment (detection bias)	High risk of bias	Subjective pain outcome ('change in restricted mouth opening' was more objective but unclear whether measured by a blinded assessor)
Incomplete outcome data (attrition bias)	Low risk of bias	Low (8%) overall attrition and fairly equally distributed
Selective reporting (reporting bias)	Low risk of bias	No evidence of selective reporting
Other bias	Low risk of bias	No other apparent bias
**Giannakopoulos 2016 [17]**		
**Characteristics**		
Study details	**Trial design:** parallel (3 arms)<br>**Location:** University clinic, Heidelberg, Germany	

	**Number of centres:** 1<br>**Recruitment period:** 2009 to 2011<br>**Sample size calculation:** no (only post-hoc to estimate sample size required for future trials)<br>**Funding:** not reported<br>**Declarations/conflicts of interest:** "The authors report no conflicts of interest"<br>* Authors provided unpublished data
Participants	**Diagnosis:** painful non-chronic (i.e. non-dysfunctional) TMD-related pain, diagnosed by use of the RDC/TMD - patients with a graded chronic pain status (GCPS) value of 3 or 4, indicative of disabling chronic pain, were not eligible for the study<br>**Duration since presenting condition began:** pain duration mean 42.98 weeks (SD 51.33)<br>**Age at baseline (years):** overall mean 41.58 (SD 16.68) - not reported by group<br>**Gender:** Gp A: 50% male; Gp B: 33.3% male; Gp C: 8.3% male<br>**Number randomised:** 36 (Gp A: 12; Gp B: 12; Gp C: 12)<br>**Number evaluated:** 36 (Gp A: 12; Gp B: 12; Gp C: 12)
Interventions	**Comparison:** 1) splint vs no splint for TMD; 2) custom-made splint vs prefabricated splint for TMD<br>All patients received counselling: their disease and its multifactorial aetiology were explained, and they were given advice on how to reduce stress on their masticatory system by avoiding extreme movements of the jaw (e.g. yawning) and by avoiding chewing hard food or chewing gum. All patients in extreme pain were allowed to use common over-the-counter analgesics, the type, amount and frequency of which were to be reported on recall<br>**Gp A:**<br>• Splint type: custom vacuum-formed oral splint fabricated on the patient's study casts in a dental laboratory<br>• Upper jaw/lower jaw: not reported<br>• Material: soft (1.5 mm thick co-polyester film)<br>• Teeth coverage: full

	• Details of impression taking: "custom alginate impressions of both dental arches and bite registrations were obtained from all patients"<br>• Instructions to patients: as above<br>• Monitoring of patients: as above<br>**Gp B:**<br>• Splint type: prefabricated oral splint with water-filled elastic pads (Aqualizer)<br>• Upper jaw/lower jaw: not reported<br>• Material: soft (water-filled elastic pads)<br>• Teeth coverage: full<br>• Details of impression taking: not used for this group<br>• Instructions to patients: wear splint during sleep and for at least 6 hours per day<br>• Monitoring of patients: none as intervention was only used for 2 weeks<br>**Gp C:** waiting-list group, received normal counselling (described above) followed by a Michigan-type hard acrylic oral splint after 2 weeks (i.e. after the study finished)<br>**Duration of treatment:** 2 weeks
Outcomes	Assessed at 2 weeks: grouped under 0 to 3 month analysis<br>**Primary:**<br>• Pain: current pain intensity using a 0 (no pain) to 10 (worst pain) NRS; we converted this to a 0 to 100 scale as reported in the majority of other studies<br>**Secondary:**<br>• Change in restricted mouth opening: unassisted maximum jaw opening (mm) (only reported mean & SD for overall sample - author provided data for opening with no pain, with pain and assisted opening; we used opening with no pain)

Risk of bias

Random sequence generation (selection bias)	Low risk of bias	“A statistician not involved in the study had provided consecutively numbered sealed envelopes with one random assignment in each"<br>Comment: probably done considering allocation concealment was done properly
Allocation concealment (selection bias)	Low risk of bias	"A statistician not involved in the study had provided consecutively numbered sealed envelopes with one random assignment in each. The envelopes were opened in sequence by the principal investigator after

		an eligible patient had given his/her written informed consent to participation in the study and had been examined"<br>Comment: the next assignment was adequately concealed from the person randomising patients
Blinding of participants and personnel (performance bias)	High risk of bias	Unable to blind patients
Blinding of outcome assessment (detection bias)	High risk of bias	Subjective pain outcome assessment by patients (but 'change in restricted mouth opening' was objective and measured by a blinded assessor)
Incomplete outcome data (attrition bias)	Low risk of bias<br>Low risk of bias	All randomised patients were included in the analyses
Selective reporting (reporting bias)	Low risk of bias	Pain outcome fully reported and author provided mean and SD for each group for the outcome of maximum mouth opening
Other bias	Low risk of bias	No apparent other bias

**Gomes 2014** [47]	
**Characteristics**	
Study details	**Trial design:** parallel (4 arms)<br>**Location:** Nove de Julho University, Sao Paulo, Brazil<br>**Number of centres:** 1<br>**Recruitment period:** June 2011 to December 2012

	**Sample size calculation:** yes (met - not powered on any of the relevant outcomes from our review)<br>**Funding:** "This study had no financial support"<br>**Declarations/conflicts of interest:** "The authors declare that they have no competing interests"
Participants	**Diagnosis:** severe TMD and sleep bruxism: 1) the Fonseca Patient History Index was used to diagnose the presence and intensity of TMD; 2) those with incisal and/or occlusal tooth wear and clinical signs in the buccal mucosa and tongue of clenching or grinding were diagnosed with bruxism based on the criteria of the American Academy of Sleep Medicine and a positive self-report of awake bruxism<br>**Duration since presenting condition began:** at least one year<br>**Age at baseline (years):** (inclusion was 18 to 40) Gp A: mean 26 (SD 3); Gp B: mean 29 (SD 4)<br>**Gender:** Gp A: 7% male; Gp B: 13% male<br>**Number randomised:** 30 (Gp A: 15; Gp B: 15)<br>**Number evaluated:** 30 (Gp A: 15; Gp B: 15)
Interventions	**Comparison:** splint vs no splint for TMD and bruxism<br>All patients in groups A and B received massage: three weekly 30-minute sessions of massage therapy performed by a physiotherapist who had undergone a training exercise for the administration of sliding and kneading manoeuvres of the masseter and anterior temporal muscles, bilaterally, over 4 consecutive weeks (total: 12 sessions)<br>**Gp A:**<br>• Splint type: custom Michigan-type occlusal splint<br>• Upper jaw<br>• Material: hard (acrylic)<br>• Teeth coverage: full<br>• Details of impression taking: the upper arch of each volunteer was moulded with irreversible hydrocolloid<br>• Instructions to patients: wear the splint while sleeping<br>• Monitoring of patients: adjustments made after 2 weeks by the same dentist in charge of the evaluation and splint fabrication<br>**Gp B:** no other treatment<br>**Gp C*:** custom Michigan-type occlusal splint (not combined with massage)

	**Gp D*:** custom silicone occlusal splint (not combined with massage)<br>* Groups C and D are excluded from this review as it was not possible to make any eligible pairwise comparisons using them<br>**Duration of treatment:** 4 weeks	
Outcomes	The outcomes measured at 4 weeks (electromyographic analysis of the masseter and anterior temporal muscles, reported as median frequency, and the Fonseca Patient History Index) were not outcomes of this review and therefore there were no usable data in this study	
**Risk of bias**		
Random sequence generation (selection bias)	Low risk of bias	"Block randomization was employed and opaque envelopes were used to conceal the allocation"<br>Comment: probably done
Allocation concealment (selection bias)	Low risk of bias	"Block randomization was employed and opaque envelopes were used to conceal the allocation"<br>Comment: probably done
Blinding of participants and personnel (performance bias)	High risk of bias	Unable to blind patients
Blinding of outcome assessment (detection bias)	Low risk of bias	Irrelevant as there are no outcomes of use for this review
Incomplete outcome data (attrition bias)	Low risk of bias	All randomised patients appear to have been included in the analyses (from correspondence with authors)
Selective reporting (reporting bias)	High risk of bias	We would expect to see pain reported in the assessment of TMD patients

Other bias	Low risk of bias	No apparent other bias

**Gomes 2015** [45]	
**Characteristics**	
Study details	**Trial design:** parallel (4 arms)<br>**Location:** Nove de Julho University, Sao Paulo, Brazil<br>**Number of centres:** 1<br>**Recruitment period:** not reported<br>**Sample size calculation**: not reported<br>**Funding:** not reported<br>**Declarations/conflicts of interest:** not reported
Participants	**Diagnosis:** sleep bruxism diagnosed by experienced dentist based on criteria of the International Classification for Sleep Disorders of the American Academy of Sleep Medicine, self-reported awake bruxism, and a minimum pain intensity score of 3 on a 11-point numerical rating scale (NRS)<br>**Duration since presenting condition began:** (months) Gp A: mean 18.16 (SD 9.33); Gp B: mean 23.19 (SD 4.84); Gp C: mean 27.55 (SD 9.41); Gp D: mean 22.94 (SD 5.02)<br>**Age at baseline (years):** (inclusion was 18 to 40) Gp A: mean 24.40 (SD 4.10); Gp B: mean 25.72 (SD 6.20); Gp C: mean 28.60 (SD 4.20); Gp D: mean 24.40 (SD 4.10)<br>**Gender:** all female<br>**Number randomised:** 100 (Gp A: 25; Gp B: 25; Gp C: 25; Gp D: 25)<br>**Number evaluated:** 78 (Gp A: 19; Gp B: 19; Gp C: 23; Gp D 17)
Interventions	**Comparison:** splint vs no splint for bruxism<br>We split the four groups/arms into two pairwise comparisons of A vs B and C vs D<br>**Gp A:**<br>• Splint type: custom Michigan-type occlusal splint<br>• Upper jaw<br>• Material: hard (acrylic)<br>• Teeth coverage: full

	• Details of impression taking: the upper arch of each volunteer was moulded with irreversible hydrocolloid<br>• Instructions to patients: wear splint while sleeping<br>• Monitoring of patients: adjustments made after 2 weeks by the same dentist in charge of the evaluation and splint fabrication<br>**Gp B:** no treatment<br>**Gp C:** combined (splint + massage) - as Gp A and Gp D<br>**Gp D:** massage: three weekly 30-minture sessions of massage of the muscles of mastication over 4 consecutive weeks (total: 12 sessions). Massage therapy performed by a physiotherapist who had undergone a training exercise for the administration of the protocol, involving sliding and kneading manoeuvres on the masseter and temporal muscles<br>**Duration of treatment:** 4 weeks
Outcomes	Assessed at 4 weeks<br>**Primary:**<br>• Pain: current pain intensity using a 0 (no pain) to 10 (worst pain) NRS<br>**Secondary:**<br>• Quality of life (including physical and emotional function): Medical Outcomes Study Short Form-36 (SF-36) - questionnaire with 36 items distributed across eight subscales: physical functioning (10 items), role physical (4 items), bodily pain (2 items), general health state (5 items), vitality (4 items), role social (2 items), role emotional (3 items) and mental health (5 items) - each reported separately apart from 'bodily pain' which was not assessed or reported (0 to 100, higher = better health) (data not usable - no SD/SE/P-values)

Risk of bias

Random sequence generation (selection bias)	Low risk of bias	"Randomization was performed using opaque envelopes containing information stipulating to which group each participant belonged"<br>Comment: probably done
Allocation concealment (selection bias)	Low risk of bias	"Randomization was performed using opaque envelopes containing information stipulating to which group each participant belonged"<br>Comment: probably done

Blinding of participants and personnel (performance bias)	High risk of bias	Unable to blind patients
Blinding of outcome assessment (detection bias)	High risk of bias	Subjective outcomes assessment by patients
Incomplete outcome data (attrition bias)	High risk of bias	Overall attrition was 22% and also differed by group (Gp A: 24%; Gp B: 24%; Gp C: 8%; Gp D: 32%). High attrition for such a short-term study
Selective reporting (reporting bias)	High risk of bias	No typical bruxism outcomes measured or reported
Other bias	Low risk of bias	No apparent other bias

**Haketa 2010** [38]	
**Characteristics**	
Study details	**Trial design:** parallel (2 arms)<br>**Location:** TMJ Clinic of the Tokyo Medical and Dental University, Japan<br>**Number of centres:** 1<br>**Recruitment period:** January to December 2006<br>**Trials registry ID**: NCT00936338<br>**Sample size calculation:** yes (not met)<br>**Funding:** public (supported by the Dental Hospital and the Department of Temporomandibular joint and Occlusion of Tokyo Medical and Dental University)<br>**Declarations/conflicts of interest**: not reported<br>* We emailed authors for data but none provided so far

Participants	**Diagnosis:** anterior disc displacement without reduction - confirmed by MRI; must have mouth-opening pain on TMJ-affected side and maximum mouth opening < 40 mm<br>**Duration since presenting condition began:** over 2 weeks<br>**Age at baseline (years):** Gp A: mean 38.6 (SD 13.8); Gp B: mean 38.8 (SD 15.2)<br>**Gender:** Gp A: 16% male; Gp B: 0% male<br>**Number randomised:** 52 (Gp A: 28; Gp B: 24)<br>**Number evaluated:** 44 (Gp A: 25; Gp B: 19)
Interventions	**Comparison:** splint v minimal treatment (exercise) for TMD<br>Instructions to all participants in both groups: all participants received a verbal explanation of the pathological conditions based on x-ray and MRI findings, and a general self-care protocol such as good posture, soft diet, teeth apart, etc. All participants were prescribed a non-steroidal anti-inflammatory drug three times every day<br>**Gp A:**<br>• Splint type: custom occlusal stabilisation splint<br>• Upper jaw<br>• Material: hard (1.5 mm thick, hard clear acrylic sheet)<br>• Teeth coverage: full<br>• Details of impression taking: vacuum-adapted to the maxillary cast<br>• Instructions to patients: information as above; splint was worn at night<br>• Monitoring of patients: not reported<br>**Gp B:** exercise intervention: manual jaw-opening exercises performed by the participants as follows: as a warm-up, the individual placed their fingertips on the edge of the mandibular anterior teeth and slowly pulled the mandible down until pain occurred on the TMJ-affected side. This mouth-opening position was held for 30 secs. Three cycles of this stretching movement were defined as a single set. The participants performed 4 sets per day, one after each meal and one after bathing<br>**Duration of treatment:** 8 weeks
Outcomes	Assessed at 4 and 8 weeks: we used the 8 week data for our 0 to 3 month analysis<br>**Primary:**

	• Pain: current maximum daily pain intensity using a 0 (no pain) to 100 (worst pain) mm VAS (no description of how measured)<br>• Harms/adverse effects: reported narratively ("No significant adverse effect was reported resulting from either treatment")<br>**Secondary:**<br>• Change in restricted mouth opening: maximum mouth-opening range (distance between the incisal edges of the upper and lower central incisors in mm) was reported separately with and without pain (we used opening without pain)<br>• Quality of life (including physical and emotional function): pain-related limitation of daily functions assessed using the "Limitation of Daily Functions for the TMD Questionnaire" - 10 questions scored using a 5-level numeric rating scale from (i) no problem at all to (v) extremely difficult. The summary score of the 10 questions ranges from 10 to 50 (data not used - median and IQR)

**Risk of bias**		
Random sequence generation (selection bias)	Low risk of bias	"The assignment was made by a table of random sampling numbers" and "a clinician drew a sealed envelope from a series of envelopes, each containing a card indicating either of two treatments for that individual"<br>Comment: appropriate method
Allocation concealment (selection bias)	Low risk of bias	"a clinician drew a sealed envelope from a series of envelopes, each containing a card indicating either of two treatments for that individual" and "One examiner who was completely independent of the treatment of participants prepared this procedure"<br>Comment: these methods should ensure that the next assignment was adequately concealed from the person randomising patients
Blinding of participants and personnel (performance bias)	High risk of bias	Unable to blind patients

Blinding of outcome assessment (detection bias)	High risk of bias	Subjective outcomes assessment by patients (except for 'change in restricted mouth opening' which was objective and measured by a blinded assessor)
Incomplete outcome data (attrition bias)	Low risk of bias	Overall attrition was 22% (Gp A: 11%; Gp B: 21%) - probably not sufficient to cause serious bias
Selective reporting (reporting bias)	Low risk of bias	No evidence of selective reporting
Other bias	Low risk of bias	No apparent other bias

**Hasanoglu 2017 [18]**	
**Characteristics**	
Study details	**Trial design:** parallel (2 arms)<br>**Location:** Department of Oral Surgery, Gazi University, Turkey<br>**Number of centres:** 1<br>**Recruitment period:** January to June 2014<br>**Sample size calculation:** yes (met)<br>**Funding:** "The authors have no support or funding to report"<br>**Declarations/conflicts of interest:** "The authors have stated explicitly that there are no conflict of interests in connection with this article"
Participants	**Diagnosis:** myofascial pain (RDC/TMD Group I: pain or ache in the jaw, temples, face, pre-auricular area or inside the ear at rest or during function and pain in response to palpation of ≥ 3 of the specified 20 muscle sites. In addition, at least one site must be ipsilateral to the site of pain complaint)<br>**Duration since presenting condition began:** Gp A: mean 3.49 years (SD 2.75); Gp B: mean 1.16 years (SD 1.36)<br>**Age at baseline (years):** (inclusion was 18+) Gp A: mean 24.6 (SD 9.2); Gp B: mean 32.25 (SD 11.97)<br>**Gender:** Gp A: 20% male; Gp B: 15% male

<table>
<tr><td></td><td colspan="2">Number randomised: 40 (Gp A: 20; Gp B: 20)
Number evaluated: 40 (Gp A: 20; Gp B: 20)</td></tr>
<tr><td>Interventions</td><td colspan="2">Comparison: splint vs no splint for TMD
Both groups received first line therapy for facial pain: guidance, assurance, counselling and behavioural changes (no further description given)
Gp A:
• Splint type: custom nociceptive trigeminal inhibition-tension suppression system (NTI-tss)
• Upper jaw/lower jaw: not reported
• Material: (hard) "For its adjustment, the thermoplastic material provided in the box with the splint is melted in hot water, filled into the concave region of the splint and adapted to lower or upper incisor teeth. The material re-polymerises again, becomes rigid, fits to the anterior teeth and avoids contact of canines and molars"
• Teeth coverage: partial
• Details of impression taking: not reported
• Instructions to patients: wear device overnight
• Monitoring of patients: not reported
Gp B: no other treatment
Duration of treatment: 6 weeks</td></tr>
<tr><td>Outcomes</td><td colspan="2">Assessed at 3 and 6 weeks: we used the 6 week data for our 0 to 3 month analysis
Primary:
• Pain: current pain intensity 0 (no pain) to 100 (worst pain) mm VAS
Secondary:
• Change in restricted mouth opening: self-assessment of functional limitation of jaw using 0 (no limitation) to 100 (severe limitation) mm VAS</td></tr>
<tr><td colspan="3">Risk of bias</td></tr>
<tr><td>Random sequence generation (selection bias)</td><td>Unclear risk of bias</td><td>"Patients were randomly divided into two groups"
Comment: insufficient information</td></tr>
<tr><td>Allocation concealment (selection bias)</td><td>Unclear risk of bias</td><td>"Patients were randomly divided into two groups"
Comment: insufficient information</td></tr>
</table>

Blinding of participants and personnel (performance bias)	High risk of bias	Unable to blind patients
Blinding of outcome assessment (detection bias)	High risk of bias	Subjective outcomes assessment by patients
Incomplete outcome data (attrition bias)	Low risk of bias	All randomised patients were included in the analyses
Selective reporting (reporting bias)	Low risk of bias	No evidence of selective reporting
Other bias	Low risk of bias	No apparent other bias

**Johansson 1991** [34]	
**Characteristics**	
Study details	**Trial design:** parallel (3 arms)<br>**Location:** Department of Stomatognathic Physiology, University of Gothenberg, Sweden<br>**Number of centres:** 1<br>**Recruitment period:** not reported<br>**Sample size calculation:** not reported<br>**Funding:** not reported<br>**Declarations/conflicts of interest:** not reported
Participants	**Diagnosis:** craniomandibular disorder (CMD): a history including signs and symptoms of CMD; complaints of headache and/or facial pain; clinical examination demonstrating tenderness to palpation in the masticatory muscles; exclusion of individuals with psychologic/psychogenic factors, trauma, surgery, or systemic joint,

	muscle, or skin diseases influencing the symptoms; exclusion of pathologic conditions in TMJs, facial skeleton, or teeth using radiographs<br>**Duration since presenting condition began:** not reported<br>**Age at baseline (years):** not reported<br>**Gender:** not reported<br>**Number randomised:** 30 (Gp A: 15; Gp B: 15)<br>**Number evaluated:** 30 (Gp A: 15; Gp B: 15)
Interventions	**Comparison:** splint vs no splint for TMD<br>**Gp A:**<br>• Splint type: custom occlusal splint<br>• Upper jaw<br>• Material: hard (acrylic)<br>• Teeth coverage: full<br>• Details of impression taking: not reported<br>• Instructions to patients: not reported<br>• Monitoring of patients: additional adjustments to splints were made after 2 weeks<br>**Gp B:** no treatment<br>**Gp C:** acupuncture (not eligible for this review)<br>**Duration of treatment:** splint group were examined at "3 months after treatment" but unclear if the treatment period lasted 3 months
Outcomes	Gp A assessed at 3 months, Gp B assessed at 2 months<br>**Primary:**<br>• Pain:<br>1) 0 (no pain) to 100 (worst pain) mm VAS (presented graphically with no SD - unable to use data)<br>2) subjective dysfunction score on 5-point scale: 1 = no pain; 2 = mild pain; 3 = moderate pain; 4 = severe pain; 5 = very severe pain (no usable data - reported as incidence of different score changes)<br>3) changes in facial pain and headache: reported as incidence of impaired, unchanged, improved, symptom-free (we dichotomised the data to report the incidence of improved and symptom-free)
**Risk of bias**	

Random sequence generation (selection bias)	Unclear risk of bias	"patients meeting the above criteria were randomly divided into three groups"<br>Comment: insufficient information
Allocation concealment (selection bias)	Unclear risk of bias	"patients meeting the above criteria were randomly divided into three groups"<br>Comment: insufficient information
Blinding of participants and personnel (performance bias)	High risk of bias	Unable to blind patients
Blinding of outcome assessment (detection bias)	High risk of bias	Subjective outcomes assessment by patients
Incomplete outcome data (attrition bias)	Low risk of bias	All randomised patients were included in the analyses
Selective reporting (reporting bias)	Low risk of bias	Poor reporting but probably not done selectively
Other bias	High risk of bias	Outcomes were assessed at 3 months for the splint group but at 2 months for the control group

**Katyayan 2014** [19]	
**Characteristics**	
Study details	**Trial design:** parallel (2 arms)<br>**Location:** Department of Prosthetic Dentistry, Government Dental College and Hospital, Ahmedabad, India<br>**Number of centres:** 1<br>**Recruitment period:** not reported ("over a period of one year")<br>**Sample size calculation:** not reported

	**Funding:** not reported<br>**Declarations/conflicts of interest:** not reported
Participants	**Diagnosis:** TMD (RDC/TMD axis I)<br>**Duration since presenting condition began:** at least 6 months<br>**Age at baseline (years):** mean 34.4 (range 20 to 56) - not reported by group<br>**Gender:** 22.5% male - not reported by group<br>**Number randomised:** 80 (Gp A: 40; Gp B: 40)<br>**Number evaluated:** 80 (Gp A: 40; Gp B: 40)
Interventions	**Comparison:** splint vs no splint for TMD<br>All patients received counselling and masticatory muscle exercises: mandible held in the maximal position for a few seconds on each movement (laterotrusive and protrusive), then with resistance from the patient's fingers. After jaw exercised, the patients were suggested to open the jaw wide stretching it with their fingers a few times for 10 to 20 seconds. Movements were repeated 7 to 10 times per training session and sessions were performed 2 to 3 times per day. Patients received written instructions and the movements were demonstrated by the dentist before treatment and after if necessary<br>**Gp A:**<br>• Splint type: custom occlusal stabilisation splint<br>• Upper jaw<br>• Material: hard (acrylic)<br>• Teeth coverage: full<br>• Details of impression taking: not reported<br>• Instructions to patients: wear at night whilst sleeping for a minimum of 12 hours. The appliance was adjusted at regular intervals and after 10 weeks, the patients were advised to gradually reduce wear of the appliance to a minimum of 8 hours per day<br>• Monitoring of patients: adjustments at 1, 7, 15, 30, 90, 150 and 180 day intervals for follow-up<br>**Gp B:** no other treatment<br>**Duration of treatment:** 6 months
Outcomes	Assessed at 6 months: grouped under > 3 to 6 months analysis<br>**Primary:**<br>• Pain:

	1) current pain intensity on 0 (no pain) to 100 (worst pain) mm VAS (authors confirmed that these scores were accidentally reported in cm - we converted them to mm)<br>2) number of painful muscle sites on palpation (out of 20 sites); 2 lb of pressure for extraoral muscles, 1 lb of pressure on the joints and intraoral muscles<br>**Secondary:**<br>• Change in restricted mouth opening: maximum mouth opening in mm - the sum of unassisted maximal interincisal opening and the vertical incisal overlap	
**Risk of bias**		
Random sequence generation (selection bias)	Low risk of bias	"The assignment was made by a table of random sampling numbers"<br>Comment: appropriate method
Allocation concealment (selection bias)	Low risk of bias	"a clinician drew a sealed envelope from a series of envelopes, each containing a card indicating either of two treatments for that individual" and "This allocation was done by a clinician who was independent of the trial and unaware of patient diagnosis, and was not involved at any stage in the clinical treatment phase"<br>Comment: these methods should ensure that the next assignment was adequately concealed from the person randomising patients
Blinding of participants and personnel (performance bias)	High risk of bias	Unable to blind patients
Blinding of outcome assessment (detection bias)	High risk of bias	Subjective outcomes assessment by patients (except for 'change in restricted mouth opening' which was objective and measured by a blinded assessor)

Incomplete outcome data (attrition bias)	Low risk of bias	All randomised patients were included in the analyses
Selective reporting (reporting bias)	Low risk of bias	No evidence of selective reporting
Other bias	Low risk of bias	No apparent other bias

Leeson 2007 [40]

Characteristics

Study details	**Trial design:** parallel (4 arms)<br>**Location:** Eastman Dental Hospital, London, UK<br>**Number of centres:** 1<br>**Recruitment period:** unclear but appears to be 1995 to 1997<br>**Sample size calculation:** yes (met)<br>**Funding:** public and pharmaceutical (medication donated by Lilly Pharmaceutical Company and the project was funded by a Department of Health Grant and locally organised research funding)<br>**Declarations/conflicts of interest:** not reported
Participants	**Diagnosis:** chronic TMD of recent onset (of more than 3 months duration, hence exposed to minimal treatment intervention) - pain in one or both TMJs with or without 1) clicking, 2) limited mouth opening, 3) muscle tenderness<br>**Duration since presenting condition began:** at least 3 months<br>**Age at baseline (years):** Gp A: mean 34.1 (SD 9.99), range 16 to 55; Gp B: mean 29.8 (SD 7.99), range 16 to 55<br>**Gender:** Gp A: 21.0% male; Gp B: 23.8% male<br>**Number randomised:** 125 (Gp A: 62; Gp B: 63)<br>**Number evaluated:** 125 (Gp A: 62; Gp B: 63) inputational analysis used (last score brought forward)
Interventions	**Comparison:** splint vs no splint for TMD

	All patients in groups A and B received medication: SSRI fluoxetine, Prozac. Initial 20mg daily, then doubled to 40mg at the two month review. After 3 months, patients who improved on medical therapy and wished to continue on treatment, remained on medication, usually at the 40mg dosage. Where pain had failed to respond, or worsened, patients were reassessed and in some cases withdrawn from continuation in the study. Further data was collected from these patients to include in the ITT analysis. All patients requested to only embark on minimal essential dental treatment and refrain from alternative pain therapies during treatment<br>**Gp A:**<br>• Splint type: custom occlusal stabilisation splint (Michigan splint)<br>• Upper jaw<br>• Material: hard (acrylic)<br>• Teeth coverage: full<br>• Details of impression taking: an appointment was arranged for impressions, wax bite and face bow recordings with the restorative lecturer. The work was then sent to Kurban Dental laboratories for construction of splint<br>• Instructions to patients: not reported<br>• Monitoring of patients: reviewed after 2 weeks for further adjustment and then minor alterations at monthly intervals up to 3 months<br>**Gp B:** no other treatment<br>**Gp C*:** splint alone (no medication)<br>**Gp D*:** placebo medication<br><br>* Groups C and D are excluded from this review as it was not possible to make any eligible pairwise comparisons using them<br>**Duration of treatment:** 3 months
Outcomes	Assessed at 1, 2 and 3 months: we used the 3-month data for our 0 to 3 month analysis<br>**Primary:**<br>• Pain:<br>1) current pain intensity on 0 (no pain) to 10 (worst pain) cm VAS (we converted this to mm in order to combine with data from other studies); this was also reported as incidence of both 25% and 50% reduction in

	VAS pain score at 3 months (we used the 50% reduction data as this enabled pooling with other data)<br>2) current pain intensity reported categorically as follows: none, mild, moderate, severe (we only used the VAS data)<br>3) pain frequency reported categorically as follows: never, occasionally, often, always (we only used the VAS data)<br>4) pain response reported categorically as follows: worse, in pain, improved, pain free (we only used the VAS data)<br>5) pain interference with life reported as yes or no (we only used the VAS data)<br>**Secondary:**<br>• Change in restricted mouth opening: maximum unassisted pain free mouth opening in mm (interincisal)<br>• Quality of life (including physical and emotional function): 1) Multidimensional Pain Inventory severity; 2) McGill Short Pain Questionnaire; 3) Kellner Illness Attitude Scale; 4) Beck BDI scores (no usable data - median and IQR)	
**Risk of bias**		
Random sequence generation (selection bias)	Low risk of bias	"Patients were randomly allocated to one of four groups, using the method of block randomisation"<br>Comment: probably done
Allocation concealment (selection bias)	Low risk of bias	"Randomisation was undertaken by a third party, namely a member of the administration or dental nursing staff. A sealed envelope was opened indicating group participation and recorded in a locked register"<br>Comment: these methods should ensure that the next assignment was adequately concealed from the person randomising patients
Blinding of participants and personnel (performance bias)	High risk of bias	Unable to blind patients

Blinding of outcome assessment (detection bias)	High risk of bias	Subjective outcomes assessment by patients (except for 'change in restricted mouth opening' which was objective but unclear whether measured by a blinded assessor)
Incomplete outcome data (attrition bias)	Low risk of bias	Inputational analysis used (last score brought forward) so that all randomised patients were included in the analyses
Selective reporting (reporting bias)	Low risk of bias	No evidence of selective reporting
Other bias	Low risk of bias	No apparent other bias

**List 1992** [35]	
**Characteristics**	
Study details	**Trial design:** parallel (3 arms)<br>**Location:** Department of Stomatognathic Physiology, University of Gothenberg, Sweden<br>**Number of centres:** 1<br>**Recruitment period:** April 1987 to March 1989<br>**Sample size calculation:** not reported<br>**Funding:** public (Jonkoping County Council and Swedish Medical Research Council, project 55)<br>**Declarations/conflicts of interest:** not reported
Participants	**Diagnosis:** craniomandibular disorder (CMD): signs and symptoms of CMD of primarily muscular origin; pain for more than 6 months; clinical dysfunction index of Di II or more according to Helkimo 1974<br>**Duration since presenting condition began:** pain for more than 6 months - median duration in years (range): Gp A: 3.0 (14.5); Gp B: 4.3 (24.5)<br>**Age at baseline (years):** Gp A: mean 39 (SD 11); Gp B: mean 48 (SD 13)<br>**Gender:** Gp A: 35% male; Gp B: 3% male

	**Number randomised:** 70 (Gp A: 40; Gp B: 30)<br>**Number evaluated:** 56 (Gp A: 34; Gp B: 22)
Interventions	**Comparison:** splint v no splint for TMD<br>**Gp A:**<br>• Splint type: custom occlusal stabilisation splints<br>• Upper jaw (only applied in the mandible area for patients with loss of molar support; n = 3)<br>• Material: hard (acrylic)<br>• Teeth coverage: full<br>• Details of impression taking: not reported<br>• Instructions to patients: used a night until evaluation seven to eight weeks later<br>• Monitoring of patients: splints were checked and adjusted after one week<br>**Gp B:** no treatment (3-month wait list)<br>**Gp C:** acupuncture (not eligible for this review)<br>**Duration of treatment:** Gp A: 6 to 8 weeks (but preceded by 1-month pre-treatment period); Gp B: on waiting list for 3 months
Outcomes	Gp A assessed at 2 months, Gp B assessed at 3 months: grouped under 0 to 3 months analysis<br>There was also 6-month and 12-month assessments but they are not reported<br>**Primary:**<br>• Pain:<br>1) 0 (no pain) to 100 (worst pain) mm VAS; recorded 3 times daily (morning, noon, evening) in a pain diary, with the average calculated on a weekly basis (appears to be presented in the study report as cm - we converted this to a mm scale)<br>2) frequency of pain: number of occasions during a week with a VAS pain score > 0, so the number of recordings during the week (3 x 7) could vary in the range 0 to 21 (we only used the VAS data above)

Risk of bias

Random sequence generation (selection bias)	Unclear risk of bias	"randomly assigned"<br>Comment: insufficient information

Allocation concealment (selection bias)	Unclear risk of bias	"randomly assigned"<br>Comment: insufficient information
Blinding of participants and personnel (performance bias)	High risk of bias	Unable to blind patients
Blinding of outcome assessment (detection bias)	High risk of bias	Subjective outcomes assessment by patients
Incomplete outcome data (attrition bias)	Low risk of bias	Overall attrition 20% (Gp A: 15%; Gp B: 27%). There were no drop-outs in the study but only pain diaries in which > 70% of the required recordings had been completed were included in the analysis. Unlikely to change the results much
Selective reporting (reporting bias)	High risk of bias	The assessments at 6 and 12 months are reported in a separate study report, but only for groups A and C
Other bias	High risk of bias	a) outcomes were assessed at 6 to 8 weeks for the splint group but at 3 months for the control group; b) substantial gender imbalance between groups (potentially indicating that the randomisation process was inadequate or did not work)

Lundh 1985 [41]

Characteristics	
Study details	**Trial design:** parallel (3 arms)<br>**Location:** Department of Stomatology, University of Lund, Sweden<br>**Number of centres:** 1<br>**Recruitment period:** January 1982 to March 1984<br>**Sample size calculation:** not reported

	**Funding:** public and industry i.e. private healthcare company (financial support from University of Lund, and Praktikertjanst AB, Sweden; study supported by Magnus Bergvalls Foundation, Torsten and Ragnar Soderbergs Foundations, and Swedish Medical Research Council)<br>**Declarations/conflicts of interest:** not reported
Participants	**Diagnosis: "**1704 patients referred for pain and dysfunction of the masticatory system", every third patient given an appointment (568). These were then subdivided into those with reciprocal clicking (clicking on opening and closing) (88) these were then subdivided again into those that could eliminate clicking by beginning mandibular movements in a position anterior to intercuspal position (centric occlusion), but not as far as edge to edge incisal potion and only these added to the trial (78). Those that could not eliminate clicking unless mandibular movements were started from edge to edge incisal position, these were excluded from the trial (10)<br>**Duration since presenting condition began:** not reported<br>**Age at baseline (years):** median 30, range 10 to 69 (not reported by group)<br>**Gender:** 31% male (not reported by group)<br>**Number randomised:** 70 (Gp A: 24; Gp B: 23; Gp C: 23)<br>**Number evaluated:** 70 (Gp A: 24; Gp B: 23; Gp C: 23)
Interventions	**Comparison:** splint vs no splint for TMD<br>**Gp A:**<br>• Splint type: custom anterior repositioning splint<br>• Upper jaw<br>• Material: hard<br>• Teeth coverage: full<br>• Details of impression taking: not reported<br>• Instructions to patients: wear 24 hours per day for 6 weeks then reduce over following 2 weeks starting with taking it out for 2 hours between meals<br>• Monitoring of patients: 6, 17 and 52 weeks<br>**Gp B:**<br>• Splint type: custom flat occlusal splint<br>• Upper jaw<br>• Material: hard<br>• Teeth coverage: full

	• Details of impression taking: not reported<br>• Instructions to patients: wear only at night for 6 weeks then reduce over following 2 weeks<br>• Monitoring of patients: as above<br>**Gp C:** no treatment<br>**Duration of treatment:** 6 weeks (but followed by 2 weeks of reduction in use and unclear thereafter)
Outcomes	Assessed at 6, 17 and 52 weeks: we used these in our 0 to 3 month, > 3 to 6 month, and > 6 to 12 month analyses respectively<br>**Primary:**<br>• Pain:<br>1) pain at rest, chewing and on protrusion assessed separately on 0 to 10 cm worsening VAS at each follow-up examination (if bilateral click then only the most painful side was scored) (no usable data – no means or SD)<br>2) palpation pain of muscles of mastication as described by Krogh-Poulsen 1979 (data not used – incidence reported separately for 4 different sites but was not equal at baseline)<br>**Secondary:**<br>• TMJ clicking: reciprocal clicking assessed using a stethoscope

Risk of bias

Random sequence generation (selection bias)	Unclear risk of bias	"randomly assigned"<br>Comment: insufficient information
Allocation concealment (selection bias)	Unclear risk of bias	"randomly assigned"<br>Comment: insufficient information
Blinding of participants and personnel (performance bias)	High risk of bias	Blinding not possible

Blinding of outcome assessment (detection bias)	High risk of bias	Subjective outcomes assessed by patient (except for clicking - but blinding was not mentioned)
Incomplete outcome data (attrition bias)	Low risk of bias	There did not appear to be any drop-outs
Selective reporting (reporting bias)	High risk of bias	No data reported for the VAS pain outcomes
Other bias	Low risk of bias	No other bias apparent

**Lundh 1988** [36]	
**Characteristics**	
Study details	**Trial design:** parallel (3 arms)<br>**Location:** 1) Department of Stomatology, School of Dentistry, Malmo, Sweden; 2) Department of Oral Surgery, University Hospital, Lund, Sweden<br>**Number of centres:** 2<br>**Recruitment period:** not reported<br>**Sample size calculation**: not reported<br>**Funding:** public and industry i.e. both private healthcare company and pharmaceutical company (supported by Magnus Bergvalls Foundation, University of Lund, Praktikertjanst AB, Sweden, Swedish Medical Research Council, Torsten and Ragnar Soderbergs Foundations, and the Ake Wiberg Foundation; Nycomed AB, Sweden provided contrast medium used for arthrography)<br>**Declarations/conflicts of interest**: not reported
Participants	**Diagnosis:** disk displacement with reduction - "902 consecutive patients referred for treatment of masticatory muscle or temporomandibular joint pain and dysfunction were clinically examined. 212 patients demonstrated temporomandibular joint reciprocal clicking

	defined as clicking during opening that did not occur unless it was preceded by clicking during closing. 149 of the 212 patients were excluded from the study. 105 of these had minor subjective complaints (graded as less than 5 on a visual analog scale with 0 and 10 as end points), 27 patients were not willing to participate in a scientific study, 11 patients needed mandibular protrusion anterior to the edge-to-edge incisal relationship to eliminate the clicking, 5 patients showed arthrographic evidence of disk displacement without reduction, and 1 patient was arthrographically normal. The study was therefore based on the remaining 63 patients" - confirmed by arthrography<br>**Duration since presenting condition began:** not reported<br>**Age at baseline (years):** median 24, range 13 to 74 (not reported by group)<br>**Gender:** 14% male (not reported by group)<br>**Number randomised:** 43 (Gp A: 21; Gp B: 22)<br>**Number evaluated:** 43 (Gp A: 21; Gp B: 22)
Interventions	**Comparison:** splint vs no splint for TMD<br>All patients were informed about basic anatomy and function of the TMJ, the mechanisms of clicking and locking, and the possible caused of pain<br>**Gp A:**<br>• Splint type: flat occlusal splint<br>• Upper jaw<br>• Material: hard<br>• Teeth coverage: full<br>• Details of impression taking: no information<br>• Instructions to patients: wear at night<br>• Monitoring of patients: not reported<br>**Gp B:** no other treatment<br>**Gp C:** disk-repositioning onlays (not eligible for this review)<br>**Duration of treatment:** 6 months
Outcomes	Assessed at 6 months: grouped under > 3 to 6 month analysis<br>**Primary:**<br>• Pain:

	1) pain at rest, chewing and on protrusion assessed separately on 0 to 10 cm worsening VAS at each follow-up examination (if bilateral click then only the most painful side was scored) (no usable data – no means or SD)<br>2) palpation pain of muscles of mastication as described by Krogh-Poulsen 1979 (data not used – incidence reported separately for 5 different sites but was not equal at baseline)<br>**Secondary:**<br>• TMJ clicking: reciprocal clicking assessed using a stethoscope and/or palpation

**Risk of bias**		
Random sequence generation (selection bias)	Unclear risk of bias	"randomly assigned"<br>Comment: insufficient information
Allocation concealment (selection bias)	Unclear risk of bias	"randomly assigned"<br>Comment: insufficient information
Blinding of participants and personnel (performance bias)	High risk of bias	Blinding not possible
Blinding of outcome assessment (detection bias)	High risk of bias	Subjective outcomes assessed by patient (except for clicking - but blinding was only done for around half of the assessments)
Incomplete outcome data (attrition bias)	Low risk of bias	There did not appear to be any drop-outs
Selective reporting (reporting bias)	High risk of bias	Pain at rest was not reported

Other bias	Low risk of bias	No other bias apparent

**Lundh 1992** [37]	
**Characteristics**	
Study details	**Trial design:** parallel (2 arms)<br>**Location:** Department of Stomatology, University of Lund, Malmo, Sweden<br>**Number of centres:** 1<br>**Recruitment period:** not reported<br>**Sample size calculation:** not reported<br>**Funding:** public and industry i.e. private healthcare company (supported by grants from Praktikertjanst AB, Sweden and by the Torsten and Ragnar Soderbergs Foundations)<br>**Declarations/conflicts of interest**: not reported
Participants	**Diagnosis:** pain on chewing (> 50 on a 0 to 100 mm VAS) with arthographically documented disc displacement without reduction in one or both TMJs<br>**Duration since presenting condition began:** not reported<br>**Age at baseline (years):** mean 29, range 14 to 61 (not reported by group)<br>**Gender:** 10% male (not reported by group)<br>**Number randomised:** 51 (Gp A: 25; Gp B: 26)<br>**Number evaluated:** 51 (Gp A: 25; Gp B: 26)
Interventions	**Comparison:** splint vs no splint for TMD<br>**Gp A:**<br>• Splint type: flat occlusal splint<br>• Upper jaw<br>• Material: hard<br>• Teeth coverage: full<br>• Details of impression taking: not reported<br>• Instructions to patients: wear at night<br>• Monitoring of patients: 1 week for further adjustments and then follow-up at 6 and 12 months<br>**Gp B:** no treatment

	**Duration of treatment:** 12 months	
Outcomes	Assessed at 12 months: grouped under > 6 to 12 month analysis<br>**Primary:**<br>• Pain:<br>1) pain at rest, during chewing and on protrusion assessed using a 0 to 100 mm worsening VAS; reported categorically as pain free, improved (at least 50% reduction), unchanged or worse (we dichotomised the data as incidence of pain-free and improved vs unchanged and worse)<br>2) changes in palpatory tenderness of masseter muscle reported as better vs unchanged or worse (data not used - those with no tenderness at start and end of study were not included)	
**Risk of bias**		
Random sequence generation (selection bias)	Unclear risk of bias	"randomized"<br>Comment: insufficient information
Allocation concealment (selection bias)	Unclear risk of bias	"randomized"<br>Comment: insufficient information
Blinding of participants and personnel (performance bias)	High risk of bias	Blinding not possible
Blinding of outcome assessment (detection bias)	High risk of bias	Subjective outcomes assessed by patient
Incomplete outcome data (attrition bias)	Low risk of bias	There did not appear to be any drop-outs

Selective reporting (reporting bias)	High risk of bias	Only outcomes with statistically significant differences were reported
Other bias	Low risk of bias	No other bias apparent

**Magnusson 1999** [42]	
**Characteristics**	
Study details`	**Trial design:** parallel (2 arms)<br>**Location:** Department of Stomatognathic Physiology, The Institute for Postgraduate Dental Education, Jonkoping, Sweden<br>**Number of centres:** 1<br>**Recruitment period:** November 1993 to September 1996<br>**Sample size calculation:** not reported<br>**Funding:** not reported<br>**Declarations/conflicts of interest:** not reported
Participants	**Diagnosis:** TMD of mainly muscular origin: patients referred to specialist clinic with main subjective symptom of tension-type headache and/orofacial pain of non-neurogenic or non-dental origin<br>**Duration since presenting condition began:** pain history of at least 1 year<br>**Age at baseline (years):** Gp A: mean 32 (range 17 to 49); Gp B: mean 37 (range 16 to 67)<br>**Gender:** not reported<br>**Number randomised:** 26 (Gp A: 14; Gp B: 12)<br>**Number evaluated:** 18 (Gp A: 9; Gp B: 9)
Interventions	**Comparison:** splint vs minimal treatment for TMD<br>**Gp A:**<br>• Splint type: interocclusal stabilisation splint (Michigan style)<br>• Upper jaw<br>• Material: hard<br>• Teeth coverage: full<br>• Details of impression taking: not reported<br>• Instructions to patients: wear at night

	• Monitoring of patients: only reports that adjustments and follow-ups were made by a dentist<br>**Gp B:** jaw exercise programme - based on different jaw movements to achieve reciprocal inhibition, proprioceptive neuromuscular facilitation, and stretching - performed at least 3 times per day with each session lasting at least 2 to 3 mins; dental assistant delivered the instructions to patients and also decided upon length of time between, as well as number of, follow-ups (she also modified patients' individual programmes when necessary by adding or removing specific exercises)<br>* Patients with significant symptoms after 3 months of treatment were offered complementary treatment with the other treatment modality. Those receiving combined treatment were analysed separately (group not included in this review)<br><br>**Duration of treatment:** 6 months
Outcomes	Assessed at 3 and 6 months: we used these in our 0 to 3 month and > 3 to 6 month analyses respectively<br>**Primary:**<br>• Pain:<br>a) categorised as none, mild and severe; reported separately for TMJ pain, muscle pain and pain on movement as part of clinical dysfunction index Di (Helkimo 1974) (we dichotmised as incidence of being pain-free)<br>b) incidence of both pain when opening the mouth and pain in the face or jaws as part of a 'subjective' anamnestic dysfunction index Ai (Helkimo 1974) (not used as too similar to other pain outcomes)<br>c) Behaviour Rating scale for pain 1 (no pain) to 6 (very strong pain, totally handicapped, can't do anything) (no usable data - graphs with no SD)<br>**Secondary:**<br>• TMJ clicking: incidence of joint sounds during functional examination<br>• Change in restricted mouth opening: maximum jaw opening in mm (no usable data - no SD); also reported as incidence of having difficulty in opening the mouth wide

Risk of bias		
Random sequence generation (selection bias)	Unclear risk of bias	"randomly assigned"<br>Comment: insufficient information
Allocation concealment (selection bias)	Unclear risk of bias	"randomly assigned"<br>Comment: insufficient information
Blinding of participants and personnel (performance bias)	High risk of bias	Blinding not possible
Blinding of outcome assessment (detection bias)	High risk of bias	Subjective outcomes assessed by patient (except for clicking - but the outcome assessor was not blinded)
Incomplete outcome data (attrition bias)	Low risk of bias	Overall attrition 31% (Gp A: 36%; Gp B: 25%) - reasons mostly the same
Selective reporting (reporting bias)	Low risk of bias	No evidence of selective reporting
Other bias	Low risk of bias	No other bias apparent

**Michelotti 2012** [20]	
**Characteristics**	
Study details	**Trial design:** parallel (2 arms)<br>**Location:** Clinic for Temporomandibular Disorders and Orofacial Pain, University of Naples Federico II, Italy<br>**Number of centres:** 1

	**Recruitment period:** 9 months (dates not reported)<br>**Sample size calculation:** no (post hoc only)<br>**Funding:** not reported<br>**Declarations/conflicts of interest:** "None of the authors reported any disclosures"
Participants	**Diagnosis:** myogenous pain according to RDC/TMD categories Ia and Ib; also objective evidence of joint pathology or dysfunction; spontaneous muscle pain > 30 mm on 100 mm VAS<br>**Duration since presenting condition began:** recurrent or constant myogenous pain for > 3 months<br>**Age at baseline (years):** Gp A: mean 30 (range 20 to 53); Gp B: mean 30 (range 18 to 49)<br>**Gender:** Gp A: 29% male; Gp B: 17% male<br>**Number randomised:** 44 (Gp A: 21; Gp B: 23)<br>**Number evaluated:** 41 (Gp A: 18; Gp B: 23)
Interventions	**Comparison:** splint vs minimal treatment for TMD<br>**Gp A:**<br>• Splint type: custom occlusal stabilisation splint (Michigan)<br>• Upper jaw<br>• Material: hard<br>• Teeth coverage: full<br>• Details of impression taking: alginate impressions of both arches and an interocclusal record with a wax wafer<br>• Instructions to patients: wear only whilst sleeping<br>• Monitoring of patients: both groups seen every 3 weeks for 15 mins (assessments carried out, motivation reinforced, and splint group had any necessary adjustments)<br>**Gp B:** education - explanation of the etiology and of the good prognosis for TMD, as well as information about self-care for the jaw musculature<br>**Duration of treatment:** 3 months
Outcomes	Assessed at 3 months: grouped under 0 to 3 month analysis<br>**Primary:**<br>• Pain: current pain intensity (spontaneous muscle pain) using 0 mm (no pain) to 100 mm (worst pain) VAS; reported as change from baseline score (unable to combine change score in primary M-A using SMD; used in sensitivity analyses of studies reporting current pain intensity on VAS/NRS at 0 to 3 months)

	**Secondary:**<br>• Change in restricted mouth opening: maximal unassisted pain free opening (mm) - distance between the maxillary and mandibular incisal edges and added the overbite measurement. 'Pain free' defined as the maximum distance the participant could open their mouth without experiencing any additional pain and discomfort; reported as change score	
**Risk of bias**		
Random sequence generation (selection bias)	Low risk of bias	"We assigned the patients to two treatment groups by means of a balanced block randomization"<br>Comment: probably done
Allocation concealment (selection bias)	Unclear risk of bias	"We assigned the patients to two treatment groups by means of a balanced block randomization"<br>Comment: insufficient information
Blinding of participants and personnel (performance bias)	High risk of bias	Blinding not possible
Blinding of outcome assessment (detection bias)	High risk of bias	Subjective outcomes assessment by patients (except for 'change in restricted mouth opening' which was objective and measured by a blinded assessor)
Incomplete outcome data (attrition bias)	Low risk of bias	Overall attrition 7% (Gp A: 14%; Gp B: 0%) - only 3 participants dropped out in Gp A so probably not enough to bias the results in a meaningful way
Selective reporting (reporting bias)	Low risk of bias	Outcomes fully reported
Other bias	Low risk of bias	No other bias apparent
**Nagata 2015 [21]**		

Characteristics	
Study details	**Trial design:** parallel (2 arms)<br>**Location:** Nippon Dental University, Niigata Hospital, Japan<br>**Number of centres:** 1<br>**Recruitment period:** June 2009 to July 2013<br>**Sample size calculation:** yes (met)<br>**Funding:** none<br>**Declarations/conflicts of interest:** "None of the authors received support from a corporation or any funding for this study"
Participants	**Diagnosis:** TMD (RDC/TMD axis I); RDC/TMD axis II was excluded<br>**Duration since presenting condition began:** Gp A: median 24 months (range 3 to 360); Gp B: median 24 months (range 4 to 72)<br>**Age at baseline (years):** Gp A: mean 41 (SD 19); Gp B: mean 43 (SD 18)<br>**Gender:** Gp A: 31% male; Gp B: 39% male<br>**Number randomised:** 201 (Gp A: 103; Gp B: 98)<br>**Number evaluated:** 181 (Gp A: 96; Gp B: 85)
Interventions	**Comparison:** splint vs no treatment for TMD<br><br>All patients in both groups received multimodal therapy: self-exercise of the jaw (pulled down on bilateral lower last molars with secondary fingers while opening jaw to the greatest possible extent - performed with 20 repetitions three times per day), CBT (guidance about clenching control during waking hours and coping with pain and stress), and received education about TMD self-management (i.e. a diet of soft foods, avoiding gum chewing and correcting bad posture). Participants with mouth-opening < 35 mm also underwent jaw manipulation<br>**Gp A:**<br>• Splint type: custom stabilisation splint<br>• Upper jaw<br>• Material: hard (acrylic)<br>• Teeth coverage: full<br>• Details of impression taking: not reported

	• Instructions to patients: wear whilst sleeping, but daytime use was not required<br>• Monitoring of patients: if no change of symptoms was achieved by this treatment, the splint was altered to the bruxism-controlled type to disturb the eccentric movements of the mandible with a steep obstacle located at the anterior teeth<br>**Gp B:** no other treatment<br><br>**Duration of treatment:** 10 weeks
Outcomes	Assessed at 2, 4, 6, 8 and 10 weeks: we used the 10-week data for our 0 to 3 month analysis<br>**Primary:**<br>• Pain: current orofacial pain using 0 to 10 worsening numerical rating scale (we converted to 0 to 100 scale)<br>**Secondary:**<br>• TMJ clicking: measured using 0 to 10 worsening numerical rating scale<br>• Change in restricted mouth opening: between upper and lower teeth in mm (not reported which teeth); asked to open mouth as wide as possible unassisted, even if they felt pain

Risk of bias

Random sequence generation (selection bias)	Low risk of bias	"Participants were randomly assigned to the non-splint multimodal therapy group (NS) or to the multimodal therapy plus splint group (NS+S) with block randomisation"<br>Comment: probably done
Allocation concealment (selection bias)	Unclear risk of bias	"Participants were randomly assigned to the non-splint multimodal therapy group (NS) or to the multimodal therapy plus splint group (NS+S) with block randomisation"<br>Comment: insufficient information
Blinding of participants and personnel (performance bias)	High risk of bias	Blinding not possible

Blinding of outcome assessment (detection bias)	High risk of bias	Subjective pain outcome assessment by patients (except for 'change in restricted mouth opening' and clicking which were objective - described as single blind so probably the assessors for these outcomes)
Incomplete outcome data (attrition bias)	Low risk of bias	Overall attrition 10% (Gp A: 7%; Gp B: 13%) - low attrition and similar reasons stated
Selective reporting (reporting bias)	Low risk of bias	No evidence of selective reporting
Other bias	Low risk of bias	No other bias apparent

**Niemela 2012** [22]	
**Characteristics**	
Study details	**Trial design:** parallel (2 arms)<br>**Location:** Oral and Maxillofacial Department, Oulu University Hospital, Finland<br>**Number of centres:** 1<br>**Recruitment period:** March 2008 to September 2009<br>**Sample size calculation:** yes (not met)<br>**Funding:** public (supported by the Finnish Dental Society, Apollonia and the Academy of Finland)<br>**Declarations/conflicts of interest:** "No conflict of interests are declared"
Participants	**Diagnosis:** TMD (RDC/TMD) - the patients were referred to the Oral and Maxillofacial Department, Oulu University Hospital, for treatment of TMD and had thus been suffering from relatively chronic and severe TMD<br>**Duration since presenting condition began:** not reported<br>**Age at baseline (years):** (inclusion = at least 20) Gp A: mean 43 (SD 13); Gp B: mean 44 (SD 13)<br>**Gender:** Gp A: 18% male; Gp B: 27% male

	**Number randomised:** 80 (Gp A: 39; Gp B: 41)<br>**Number evaluated:** 1 month: 76 (Gp A: 39; Gp B: 37); 1 year: 78 (Gp A: 37; Gp B: 41) - ITT ("Two patients dropped out of the trial from the splint group; one did not attend any of the check-ups and the other was offered other treatment, that is orthognathic surgery. In addition, during the 1-year follow-up, altogether 16 patients interrupted their attendance to the trial or did not show up for their appointed follow-up. Sixteen controls were transferred from the control group to the splint group because of their symptoms and need of treatment. Thirteen patients (10 patients in the splint group and three in the control group) were treated with arthrocentesis of the TMJ during the study. All the patients in the total sample were defined as belonging to the 'intention-to-treat' (ITT) population except for the two who were excluded at the beginning of the trial. Thus, the ITT also included those who switched groups or those who in whichever group received other treatment than initially planned based on the group criteria")
Interventions	**Comparison:** splint vs no treatment for TMD<br><br>All patients in both groups received counselling and instructions for masticatory muscle exercises - at the beginning of the training program, active mouth openings, laterotrusive movements and protrusive movements were performed. The mandible was held in the maximal positions for a few seconds on each movement. Thereafter, these movements were made towards resistance (using patient's own fingers). After jaw exercises, the patients were suggested to open the jaw wide, stretching it with fingers a few times for 10–20 s. These movements were repeated 7–10 times per training sessions, and the sessions were performed 2–3 times per day. The patients received written instructions, and the movements were also demonstrated by the dentist before the treatment and reprised if necessary<br>**Gp A:**<br>Splint type: custom occlusal stabilisation splint<br>• Upper jaw/lower jaw: not reported<br>• Material: hard (acrylic)

	• Teeth coverage: full<br>• Details of impression taking: "occlusion of the splint was defined in the centric relation occlusion using wax"<br>• Instructions to patients: use every night during study<br>• Monitoring of patients: not reported<br>**Gp B:** no other treatment<br><br>**Duration of treatment:** 1 year
Outcomes	Assessed at 1, 3, 6 months and 1 year (mouth opening only assessed at 1 month)<br><br>VAS pain only reported as median at 3 and 6 months so data not used<br>**Primary:**<br>• Pain:<br>a) current facial pain intensity using 0 (no pain) to 10 (worse pain) cm VAS (we converted this to mm in order to combine with data from other studies)<br>b) number of painful masticatory muscle sites on palpation (out of 20 sites) (only VAS data used - baseline scores for this outcome were not comparable)<br>c) incidence of TMJ pain on lateral or posterior palpation of one or both TMJs (only VAS data used - baseline scores for this outcome were not comparable)<br>**Secondary:**<br>• Change in restricted mouth opening: unassisted maximal opening (exact location not reported; whether with/without/until pain not reported)<br>• Quality of life (including physical and emotional function): 14-item Oral Health Impact Profile (OHIP-14) - responses were as follows: 0 = never, 1 = hardly ever, 2 = occasionally, 3 = fairly often and 4 = very often; The OHIP severity score was calculated by summing the ordinal values for 14 items (range 0 to 56)

Risk of bias

Random sequence generation (selection bias)	Low risk of bias	"Patients were assigned randomly using computer generated random number"<br>Comment: appropriate method

Allocation concealment (selection bias)	Unclear risk of bias	"Patients were assigned randomly using computer generated random number"<br>Comment: insufficient information
Blinding of participants and personnel (performance bias)	High risk of bias	Blinding not possible
Blinding of outcome assessment (detection bias)	High risk of bias	Subjective pain outcomes assessed by patients (except for 'change in restricted mouth opening' which was objective and measured by a blinded assessor)
Incomplete outcome data (attrition bias)	Low risk of bias	Low attrition and ITT was used at 1 year for pain on VAS (but quality of life data have very high attrition at all assessment points and should be considered at high risk of bias)
Selective reporting (reporting bias)	Low risk of bias	Outcomes fully reported
Other bias	Low risk of bias	No other apparent bias

**Nitecka-Buchta 2014 [23]**	
**Characteristics**	
Study details	**Trial design:** parallel (2 arms)<br>**Location:** Department of Orthodontics and TMJ Dysfunction, Medical University of Silesia Katowice, Zabrze, Poland<br>**Number of centres:** 1<br>**Recruitment period:** not reported<br>**Sample size calculation:** not reported<br>**Funding:** public (study was funded by the Medical University of Silesia Katowice, Poland)

	**Declarations/conflicts of interest:** "The authors have no conflict of interest regarding this commentary"
Participants	**Diagnosis:** RDC/TMD examination for group Ia (myofascial pain) and Ib (myofascial pain with limited opening)<br>**Duration since presenting condition began:** not reported<br>**Age at baseline (years):** overall mean 47 (range 44 to 70)<br>**Gender:** Gp A: 29% male; Gp B: 30% male<br>**Number randomised:** 72 (Gp A: 36; Gp B: 36)<br>**Number evaluated:** 65 (Gp A: 35; Gp B: 30)
Interventions	**Comparison:** splint vs no splint for TMD<br>**Gp A:**<br>• Splint type: custom occlusal splint<br>• Upper jaw/lower jaw: not reported<br>• Material: not reported<br>• Teeth coverage: not reported<br>• Details of impression taking: not reported<br>• Instructions to patients: not reported<br>• Monitoring of patients: not reported<br>**Gp B:** no treatment<br>**Duration of treatment:** 30 days
Outcomes	Assessed at 30 days: grouped under 0 to 3 month analysis<br>**Primary:**<br>• Pain: current pain intensity on 0 (no pain) to 10 (worst pain) cm VAS (we converted this to mm)<br>• Harms/adverse effects: reported narratively ("no complications or any unintended effects in either group")

Risk of bias

Random sequence generation (selection bias)	Low risk of bias	"randomised…allocated into one of two groups (by picking a colour card from an envelope)"<br>Comment: probably done
Allocation concealment (selection bias)	Low risk of bias	"One person enrolled participants in the study, and another dental practitioner assigned them to the interventions"<br>Comment: attempted to conceal allocation

Blinding of participants and personnel (performance bias)	High risk of bias	Blinding not possible
Blinding of outcome assessment (detection bias)	High risk of bias	Subjective pain outcome assessment by patients
Incomplete outcome data (attrition bias)	High risk of bias	10% attrition (Gp A: 3%; Gp B: 17%) which differed by group and may feasibly have biased results
Selective reporting (reporting bias)	Low risk of bias	Pain reported clearly
Other bias	Low risk of bias	No other apparent bias

**Pierce 1988** [46]	
**Characteristics**	
Study details	**Trial design:** parallel (5 arms)<br>**Location:** School of Dental Medicine, State University of New York, Buffalo, USA<br>**Number of centres:** 1<br>**Recruitment period:** not reported<br>**Sample size calculation:** not reported<br>**Funding:** public (study was supported in part by research grants DE-05344 and DE-04358 from the National Institutes of Health, USA)<br>**Declarations/conflicts of interest:** not reported
Participants	**Diagnosis:** 1) self-reported history of bruxism; or 2) currently bruxing and someone else had heard them bruxing; or 3) tooth wear indicating bruxism. This was then confirmed by electromyographic (EMG) activity

	and patients were only included if they had a baseline of mean bruxing episodes per hour of greater than 1.0<br>**Duration since presenting condition began:** not reported<br>**Age at baseline (years):** overall mean 38 (range 18 to 72)<br>**Gender:** 35% male<br>**Number randomised:** 40 (Gp A: 20; Gp B: 20)<br>**Number evaluated:** not reported
Interventions	**Comparison:** splint vs no splint for bruxism<br>**Gp A:**<br>• Splint type: flat-plane occlusal splint with cuspid rise<br>• Upper jaw<br>• Material: hard (acrylic)<br>• Teeth coverage: full<br>• Details of impression taking: not reported<br>• Instructions to patients: wear at night<br>• Monitoring of patients: asked to return during first week of treatment for splint adjustment, or any other time if discomfort or lack of fit was experienced<br>**Gp B:** no treatment<br>**Gp C:** "massed negative practice": individually tailored; 6 blocks of clenching per day consisting of 5 clench/relax cycles varying between 5 seconds and 1 minute; each clench continued to the point of discomfort, not pain, and then discontinued (not used due to more appropriate control group consisting of no treatment)<br>**Gp D:** nocturnal biofeedback (not eligible for this review)<br>**Gp E:** diurnal biofeedback (not eligible for this review)<br>EMG monitoring of all patients whilst sleeping (at their home i.e. not in a sleep clinic); use of EMG monitored at regular appointments<br>**Duration of treatment:** 2 weeks
Outcomes	Outcomes assessed at 2 weeks (for 2-week treatment phase) and at 6 months (EMG monitoring carried out for a 2-week period and to calculate the means for the bruxism outcomes)<br>**Primary:**<br>• Tooth wear (bruxism only): not reported<br>**Secondary:**<br>• Bruxism severity: duration of bruxing per hour (no usable data - no SD/SE/CI or P-values)

	• Bruxism frequency: episodes per hour (no usable data - no SD/SE/CI or P-values)	
**Risk of bias**		
Random sequence generation (selection bias)	Unclear risk of bias	"each subject was randomly assigned to one of the five experimental groups"<br>Comment: insufficient information
Allocation concealment (selection bias)	Unclear risk of bias	"each subject was randomly assigned to one of the five experimental groups"<br>Comment: insufficient information
Blinding of participants and personnel (performance bias)	High risk of bias	Not possible to blind
Blinding of outcome assessment (detection bias)	Low risk of bias	Objective assessment using EMG monitoring whilst participants were asleep
Incomplete outcome data (attrition bias)	Unclear risk of bias	The numbers analysed per group at each assessment were not reported
Selective reporting (reporting bias)	High risk of bias	Poor reporting of outcomes
Other bias	Low risk of bias	No other apparent bias
**Rampello 2013** [43]		
**Characteristics**		
Study details	**Trial design:** parallel (2 arms)<br>**Location:** Clinical Gnathology Service, Umberto I Polyclinic, Sapienza University, Rome, Italy	

	**Number of centres:** 1<br>**Recruitment period:** January to May 2011<br>**Sample size calculation:** not reported<br>**Funding:** not reported<br>**Declarations/conflicts of interest:** "all authors report no conflict of interest relevant to this article" - however, one of the authors designed and patented the splint (UNIRA) used in the study<br>* Emailed authors for info and data but none provided so far
Participants	**Diagnosis:** muscular, articular and headache/migraine VAS scores all > 30; nonreducing dislocations of the articular disc in acute cases of miocene; parafunctions associated with muscular and/or articular pain; limited mouth opening of muscular origin; abstract mentions "according to the RDC-TMD (SPEC) criteria"<br>**Duration since presenting condition began:** not reported<br>**Age at baseline (years):** Gp A: mean 30.9, SD 7.9 (range 20 to 46); Gp B: mean 30.2, SD 7.3 (range 20 to 45)<br>**Gender:** Gp A: 20% male; Gp B: 12% male<br>**Number randomised:** 50 (Gp A: 25; Gp B: 25)<br>**Number evaluated:** 50 (Gp A: 25; Gp B: 25)
Interventions	**Comparison:** splint vs no splint for TMD<br>**Gp A:**<br>• Splint type: UNIRA (Universal Neuromuscular Immediate Relaxing Appliance) "ready-to-use" occlusal splint<br>• Upper jaw/lower jaw: not reported<br>• Material: (soft) polyvinyl (polypropylene)<br>• Teeth coverage: not reported<br>• Details of impression taking: not reported<br>• Instructions to patients: "applied for a minimum of 1 night, followed by rest to a maximum of 12 h/day (including night and rest) for patients with intense pain"; no other form of therapy permitted<br>• Monitoring of patients: not reported<br>**Gp B:** no treatment<br>**Duration of treatment:** maximum of 3 months
Outcomes	Assessed at 3 months for splint group but 4 months for control: we would have grouped under 0 to 3 month analysis

	**Primary:**<br>• Pain: 0 to 100 VAS, separate ratings for: 1) muscular, 2) migraine, 3) cervical, 4) TMJ, reported only graphically with mean and SE but unable to accurately use; also reported for numbers cured/improved of above pains 1 to 4 (however, only some of the patients in each group had the specified pain type at baseline, and very poorly reported - not usable)<br>**Secondary:**<br>• Change in restricted mouth opening: only reported for splint group and for those who started with restricted mouth opening (data not usable)

**Risk of bias**		
Random sequence generation (selection bias)	Unclear risk of bias	"divided randomly"<br>Comment: insufficient information
Allocation concealment (selection bias)	Unclear risk of bias	"divided randomly"<br>Comment: insufficient information
Blinding of participants and personnel (performance bias)	High risk of bias	Blinding not possible
Blinding of outcome assessment (detection bias)	High risk of bias	Subjective pain outcome assessment by patients
Incomplete outcome data (attrition bias)	Low risk of bias	Does not appear to have been any attrition
Selective reporting (reporting bias)	Low risk of bias	Although there are no usable data, this is not related to selective reporting

Other bias	High risk of bias	The splint group outcomes were assessed at 3 months (end of treatment) whereas the 'no treatment' control group were assessed at 4 months

Sharma 2016 [28]

Characteristics

Study details	**Trial design:** parallel (3 arms)<br>**Location:** School of Dental Medicine, State University of New York, Buffalo, USA<br>**Number of centres:** 1<br>**Recruitment period:** not reported<br>**Sample size calculation:** no (post-hoc only)<br>**Funding:** not reported<br>**Declarations/conflicts of interest:** not reported
Participants	**Diagnosis:** bilateral masseter myalgia according Diagnostic Criteria for TMDs (DC/TMD); pain intensity of 5 or more on a 0 (no pain) to 10 (worst pain) scale; morning symptoms of jaw pain and stiffness<br>**Duration since presenting condition began:** not reported<br>**Age at baseline (years):** (overall range 24 to 62) Gp A: mean 42.6 (SD 9.6); Gp B: mean 35 (SD 9.5)<br>**Gender:** Gp A: 0% male; Gp B: 17% male<br>**Number randomised:** 13 (Gp A: 7; Gp B: 6)<br>**Number evaluated:** 13 (Gp A: 7; Gp B: 6) - two drop-outs but not reported by group A, B or C, and not clear if ITT used
Interventions	**Comparison:** splint vs no splint for TMD<br>In groups A and B, if indicated, ethyl chloride vapocoolant spray was used during spray and stretch physical therapy sessions once per week for a total of four treatment sessions<br>**Gp A:**<br>• Splint type: custom occlusal flat plane splint<br>• Upper jaw<br>• Material: hard/soft dual laminate material (a compound material made up of hard polycarbonate (PC) base material and a soft thermoplastic (TPU) material); a translucent 2.5 mm (1.2 mm PC / 1.3 mm TPU) dura-soft sheet was used<br>• Teeth coverage: full

	• Details of impression taking: not reported<br>• Instructions to patients: not reported<br>• Monitoring of patients: patients see weekly and splint checked and polished (followed by spray and stretch, as described above, if indicated)<br>**Gp B:** no other treatment<br>**Gp C:** above splint alone (this group was not included in the review as it was not possible to include it in an eligible pairwise comparison)<br>**Duration of treatment:** 5 weeks
Outcomes	Assessed at 5 weeks: grouped under 0 to 3 month analysis<br>**Primary:**<br>• Pain: Characteristic Pain Intensity (CPI) - patients scored: 1) current pain, 2) worst pain, 3) average pain each on 0 (no pain) to 10 (worst pain) scale - scores 1 to 3 were summed together, divided by 3 and then multiplied by 100 to get a score on a 0 to 100 scale; reported as change score<br>**Secondary:**<br>• Change in restricted mouth opening: 1) pain-free opening (mm); 2) maximum unassisted opening (mm); 3) maximum assisted opening (mm) (we used pain-free opening data); reported as change score<br>• Quality of life (including physical and emotional function): assessed using Axis II questionnaires: 1) Patient Health Questionnaire-9; 2) Patient Health Questionnaire-15; 3) Generalized Anxiety Disorder-7 scale (scales not described - unclear direction of benefit - data not used)

Risk of bias

Random sequence generation (selection bias)	Low risk of bias	Quote: "A computer generated spreadsheet was utilized to randomly assign each subject before recruiting any subjects, a block randomization process was performed to evenly distribute every participant into one of the three treatment arms"<br>Comment: probably done
Allocation concealment (selection bias)	Unclear risk of bias	No mention of allocation concealment
Blinding of participants and personnel	High risk of bias	Not possible to blind patients

(performance bias)		
Blinding of outcome assessment (detection bias)	High risk of bias	Subjective outcomes assessment by patients (except for 'change in restricted mouth opening' which was objective and measured by a blinded assessor)
Incomplete outcome data (attrition bias)	Unclear risk of bias	Two drop-outs but not reported which group and not clear if ITT used in analyses
Selective reporting (reporting bias)	Low risk of bias	Outcomes fully reported
Other bias	Low risk of bias	No other bias apparent

**Tatli 2017 [29]**	
**Characteristics**	
Study details	**Trial design:** parallel (3 arms)<br>**Location:** TMD clinic, Cukurova University Dental Hospital, Adana, Turkey<br>**Number of centres:** 1<br>**Recruitment period:** not reported<br>**Sample size calculation:** yes (achieved)<br>**Funding:** none<br>**Declarations/conflicts of interest:** "nothing to declare"
Participants	**Diagnosis:** unilateral TMJ disc displacement without reduction diagnosis based on clinical DC/TMD (history of reduction in mouth opening, TMJ pain during palpation and/or function, TMJ clicking) and MRI<br>**Duration since presenting condition began:** not reported<br>**Age at baseline (years):** Gp A: mean 38.9 (SD 11.3); Gp B: mean 35.2 (SD 9.4)<br>**Gender:** Gp A: 2.5% male; Gp B: 12.5% male

	**Number randomised:** 80 (Gp A: 40; Gp B: 40)<br>**Number evaluated:** 80 (Gp A: 40; Gp B: 40)
Interventions	**Comparison:** splint vs no splint for TMD<br>All patients in Gps A and B were treated with arthrocentesis plus sodium hyaluronate at the start of the study<br>**Gp A:**<br>• Splint type: occlusal stabilisation splint<br>• Upper jaw/lower jaw: not reported<br>• Material: hard (acrylic)<br>• Teeth coverage: full<br>• Details of impression taking: not reported<br>• Instructions to patients: wear at night and also for 1 to 2 hours during the day; patients in all groups instructed to use ibuprofen (600 mg) when needed<br>• Monitoring of patients: not reported<br>**Gp B:** no other treatment<br>**Gp C:** stabilisation splint alone (i.e. no arthrocentesis and sodium hyaluronate) – excluded from the review as not comparable with other groups<br>**Duration of treatment:** 6 months
Outcomes	Assessed as 1, 3 and 6 months: we used the 3 and 6 month data in our 0 to 3 month and > 3 to 6 month analyses respectively<br>**Primary:**<br>• Pain:<br>1) current pain intensity 0 to 10 cm VAS (we converted this to mm in order to combine with data from other studies)<br>2) Characteristic Pain Intensity (CPI) - patients scored: 1) current pain, 2) worst pain, 3) average pain each on 0 (no pain) to 10 (worst pain) scale - scores 1 to 3 were summed together, divided by 3 and then multiplied by 100 to get a score on a 0 to 100 scale<br>• Harms/adverse effects: reported but they were all due to arthrocentesis<br>**Secondary:**<br>• Change in restricted mouth opening: maximum mouth opening measured between the edges of the upper and lower central incisors in mm (unclear if with/without pain or assisted/unassisted)<br>• Quality of life (including physical and emotional function): pain-related disability (0 to 100 worsening scale) and psychological

	status (0 to 4 worsening scale) both separately assessed using RDC/TMD Axis II biobehavioural questionnaire

**Risk of bias**		
Random sequence generation (selection bias)	Low risk of bias	"assigned randomly to the treatment groups using randomization software"<br>Comment: appropriate method
Allocation concealment (selection bias)	Unclear risk of bias	"assigned randomly to the treatment groups using randomization software"<br><br>Comment: insufficient information
Blinding of participants and personnel (performance bias)	High risk of bias	Blinding not possible
Blinding of outcome assessment (detection bias)	High risk of bias	Subjective outcomes assessment by patients (except for 'change in restricted mouth opening' which was objective and measured by a blinded assessor)
Incomplete outcome data (attrition bias)	Low risk of bias	All randomised patients were included in the analyses
Selective reporting (reporting bias)	Low risk of bias	Outcomes fully reported
Other bias	Low risk of bias	No other bias apparent

**Tavera 2012 [24]**	
**Characteristics**	
Study details	**Trial design:** parallel (3 arms)<br>**Location:** Mexican Institute for Clinical Research, Mexico

	**Number of centres:** 1<br>**Recruitment period:** May to September 2008<br>**Trials registry ID:** NCT00815776<br>**Sample size calculation:** not reported<br>**Funding:** not reported<br>**Declarations/conflicts of interest:** not reported<br><br>* We emailed authors for data but none provided so far
Participants	**Diagnosis:** RDC/TMD diagnosis of myofascial pain, arthralgia, and/or disc displacement with reduction, and a VAS pain score of > 4 (0 to 10 worsening scale)<br>**Duration since presenting condition began:** not reported<br>**Age at baseline (years):** Gp A: mean 38 (SD 11); Gp B: mean 36.3 (SD 13)<br>**Gender:** Gp A: 17% male; Gp B: 11% male<br>**Number randomised:** 108 (Gp A: 71; Gp B: 37)<br>**Number evaluated:** 78 (Gp A: 56; Gp B: 22)
Interventions	**Comparison:** splint vs minimal treatment for TMD<br>**Gp A:**<br>• Splint type: flat-planed occlusal stabilisation splint<br>• Upper jaw/lower jaw (not reported)<br>• Material: hard (plastic)<br>• Teeth coverage: full ("full coverage" and "fits over the occlusal one-third surfaces of the dentition")<br>• Details of impression taking: not reported<br>• Instructions to patients: wear at night<br>• Monitoring of patients: not reported<br>**Gp B:** jaw exercise: patients instructed to open jaw as wide as possible without pain and hold the position for 5 seconds. Patients then closed their jaw and rested for 10 seconds. This was performed 10 times in a row. Also advised to apply warm compress to the jaw area after the exercises for 10 minutes<br>**Gp C:** TMD*es* (a novel, noninvasive and reversible custom-fit ear insert worn in the outer third of both ear canals; small, hollow and constructed from rigid, medical grade plastics used in hearing devices) (not used due to more appropriate control group consisting of jaw exercise)

	**Duration of treatment:** 3 months	
Outcomes	Assessed at 1, 2 and 3 months: we would have used the 3 month data in our 0 to 3 month analyses<br>**Primary:**<br>• Pain: 0 (no pain) to 10 (worst pain) VAS (mean and SD not reported for each group - data not usable)<br>• Harms/adverse effects: incidence of the following treatment-related adverse events: discomfort or pain, increased TMD symptoms, diminished hearing acuity, headache, dizziness or nausea, other (jaw muscle/gum-related for Gp A)<br>**Secondary:**<br>• Patient satisfaction: only reported for Gps A and C so not usable<br>• Adherence to treatment: assessed using a daily diary and average usage reported as hours per day for Gp A and C, and average exercise repetitions for Gp B; therefore data not comparable and not used	
**Risk of bias**		
Random sequence generation (selection bias)	Unclear risk of bias	"randomly assigned"<br>Comment: insufficient information
Allocation concealment (selection bias)	Unclear risk of bias	"randomly assigned"<br>Comment: insufficient information
Blinding of participants and personnel (performance bias)	High risk of bias	Blinding was not possible
Blinding of outcome assessment (detection bias)	High risk of bias	Neither patients nor study personnel were blinded
Incomplete outcome data (attrition bias)	High risk of bias	Overall attrition was 28% (Gp A: 20%; Gp B: 43% at 2 months; very similar at 3 months). Attrition was notably higher in Gp B

Selective reporting (reporting bias)	High risk of bias	Very poor reporting of outcomes - focuses on TMD*es* group (Gp C)
Other bias	Low risk of bias	No other bias apparent

**Truelove 2006 [25]**	
**Characteristics**	
Study details	**Trial design:** parallel (3 arms)<br>**Location:** Orofacial Pain Clinic, Department of Oral Medicine, University of Washington, Seattle, USA<br>**Number of centres:** 1<br>**Recruitment period:** not reported<br>**Sample size calculation:** yes (not met)<br>**Funding:** public (study supported by National Institute of Dental and Craniofacial Research grant P01 DE-08773)<br>**Declarations/conflicts of interest:** not reported
Participants	**Diagnosis:** RDC/TMD Axis I diagnosis of myofascial pain (Group Ia or Ib) with or without a concurrent diagnosis of arthralgia (Group IIIa) or disk displacement with reduction (Group IIa), as well as an RDC/TMD Axis II Graded Chronic Pain score of Grade I (low pain) or Grade II (high pain), both of which had no or minimal pain-related psychosocial interference. Any other RDC/TMD Axis<br>I diagnosis (e.g. arthritis, disk displacement without reduction) was excluded<br>**Duration since presenting condition began:** years with facial pain: Gp A: mean 6 (SD 9); Gp B: mean 5 (SD 6); Gp C: mean 5 (SD 5)<br>**Age at baseline (years):** Gp A: mean 36 (SD 11); Gp B: mean 35 (SD 12); Gp C: mean 36 (SD 11)<br>**Gender:** Gp A: 13% male; Gp B: 10% male; Gp C: 19% male<br>**Number randomised:** 200 (Gp A: 68; Gp B: 68; Gp C: 64)<br>**Number evaluated:** 3 months: 164 (Gp A: 54; Gp B: 56

<table>
<tr><td>Interventions</td><td>Comparison: 1) splint vs no splint for TMD; 2) custom-made splint vs prefabricated splint for TMD
All groups received usual treatment: dentist-prescribed, conservative and reversible self-care strategies that required the dentist to follow a standardized treatment checklist that identifies all treatment recommendations (jaw relaxation, reduction of parafunction, thermal packs, NSAIDs, passive opening stretches and suggestions about stress reduction); treatments such as narcotic analgesics, antidepressant medications and use of a non-study prescribed splint were discouraged
Gp A:
Splint type: custom flat-plane hard splint adjusted to centric occlusion
Upper jaw
Material: hard (acrylic)
Teeth coverage: full
Details of impression taking: not reported
Instructions to patients: wear at night plus two hours during the day; discontinue if problems developed
Monitoring of patients: patients in all 3 groups followed up at 3, 6 and 12 months (nothing mentioned regarding adjustment/monitoring of the actual splints)
Gp B:
Splint type: (prefabricated) soft thermoplastic athletic mouthguard splint (with the dentist supervising and directing the patient in splint fabrication)
Upper jaw
Material: soft (vinyl)
Teeth coverage: full
Details of impression taking: "we took a bite registration using dental wax to provide an oral procedure of comparable duration"
Instructions to patients: as above
Monitoring of patients: as above
Gp C: no other treatment
Duration of treatment: 12 months</td></tr>
<tr><td>Outcomes</td><td>Assessments at 3, 6* and 12 months: we used the 3 and 12 month data in our 0 to 3 month and > 6 to 12 months analyses respectively

* Data at 6 months not reported because "we typically found six-month data to be intermediate or equivalent to 12-month data"</td></tr>
</table>

	**Primary:**<br>• Pain:<br>1) characteristic pain intensity 0 to 10 scale (the mean of present, average and worst TMD-related pain in the past two months) (we converted to 0 to 100 scale; range of SDs reported - we used median value; unclear which group the single SD in the graph belongs to)<br>2) pain duration (both hours/day and days/month) (no usable data - reported narratively)<br>3) pain on palpation assessed as number of extraoral muscle sites (0 to 16), intraoral muscle sites (0 to 4) and TMJ sites (0 to 4)<br>• Harms/adverse effects: "no subjects reported an adverse effect with any of the treatments"<br>**Secondary:**<br>• TMJ clicking: on opening, closing or both; patient-assessed and clinician-assessed, reported as incidence (we used clinician-assessed in line with other studies, and also because they were blinded)<br>• Change in restricted mouth opening: vertical jaw opening in mm, reported both as unassisted without pain and assisted (no usable data - no SD reported)<br>• Adherence to treatment: reported for custom-made splint vs prefabricated splint (not clear what level of compliance e.g. using splint all the time/majority of the time/etc)	
**Risk of bias**		
Random sequence generation (selection bias)	Low risk of bias	"We generated randomization assignments using randomly selected block sizes of six, nine or 12 and stratified them by provider"<br>Comment: probably done
Allocation concealment (selection bias)	Low risk of bias	"We concealed randomization to all study personnel until after we obtained the subjects' consent"<br>Comment: randomly permuted block size, probably done adequately
Blinding of participants and personnel (performance bias)	High risk of bias	Blinding was not possible

Blinding of outcome assessment (detection bias)	High risk of bias	Subjective outcomes assessment by patients (except for 'change in restricted mouth opening' and 'TMJ clicking' which were objective and measured by a blinded assessor)
Incomplete outcome data (attrition bias)	High risk of bias	Overall attrition 18% (Gp A: 21%; Gp B: 18%; Gp C: 16%) at 3 months; overall attrition 16% (Gp A: 4%; Gp B: 19%; Gp C: 25%) at 12 months. There was a large difference between Gp A and the other groups at 12 months
Selective reporting (reporting bias)	Low risk of bias	Although we were unable to use some of the data, this does not appear to be due to selective reporting
Other bias	Low risk of bias	No other bias apparent

Wahlund 2003 [26]

Characteristics

Study details	**Trial design:** parallel (3 arms)<br>**Location:** TMD Unit, Specialist Centre for Oral Rehabilitation, Linkoping, Sweden<br>**Number of centres:** 1<br>**Recruitment period:** 1996 to 2000<br>**Sample size calculation:** not reported<br>**Funding:** public (study was supported by the Public Dental Service of Ostergotland - County Council)<br>**Declarations/conflicts of interest:** not reported
Participants	**Diagnosis:** TMD pain according to RDC/TMD<br>**Duration since presenting condition began:** at least 3 months<br>**Age at baseline (years):** overall range: 12 to 18; Gp A: mean 15.7 (SD 2.1); Gp B: mean 14.8 (SD 1.9)<br>**Gender:** Gp A: 26% male; Gp B: 31% male<br>**Number randomised:** 81 (Gp A: 42; Gp B: 39)<br>**Number evaluated:** 76 (Gp A: 37; Gp B: 39)

Interventions	**Comparison:** splint vs no splint for TMD<br>All patients received an individual 30-minute session in which TMD-related anatomy, pain epidemiology, parafunction and stress were discussed<br>**Gp A:**<br>• Splint type: occlusal stabilisation splint<br>• Upper jaw<br>• Material: not reported<br>• Teeth coverage: full<br>• Details of impression taking: not reported<br>• Instructions to patients: wear every night during treatment phase and then whenever needed until 6-month follow-up point<br>• Monitoring of patients: 4 visits at 2 week intervals (1st = brief info described above; 2nd = impression taking; 3rd = splint fitted and adjusted; 4th splint checked and readjusted)<br>**Gp B:** no other treatment<br>**Gp C:** relaxation training – this was not considered to be minimal treatment due to multiple individual sessions and was therefore excluded from this review<br>**Duration of treatment:** not clear from the text of the study report. There was a treatment period which seems to have been 2 or 4 weeks long, but then there was follow-up at 6 months. From the end of the treatment period to the 6-month follow-up, patients were instructed to wear their splint whenever needed
Outcomes	All outcomes are reported at the end of treatment period (unclear how many weeks) which we included in our 0 to 3 month analysis, and at 6 months follow-up which we included in our > 3 to 6 month analysis<br>**Primary:**<br>• Pain: not clear if all measures were recorded in the daily pain diary or at the 2 assessment timepoints:<br>1) pain intensity on 0 (no pain) to 10 (worst pain imaginable) cm VAS (unable to use data - not possible to read SDs from graph)<br>2) pain frequency on 5-point scale (never, 1 to 2 times a month, once per week, several times per week, daily) (unable to use - reported as median and quartiles)

	3) pain index on a 0 to 50 worsening scale (pain intensity (VAS) multiplied by frequency of pain) (unable to use data - not possible to read SDs from graph)<br>4) incidence of 50% reduction in pain index (unable to use data - unclear whether data are for the end of treatment or 6-month follow-up)<br>5) pressure pain threshold measured using a pressure algometer that applied pressure on the skin surface over the TMJ and masticatory muscles (scale/units of measurement not stated but higher score = better outcome)<br>• Harms/adverse effects: "None of the patients in any of the treatment modes reported any major adverse effects"<br>**Secondary:**<br>• TMJ clicking: measured but not reported<br>• Change in restricted mouth opening: reported as maximum assisted mandibular opening (mm) without pain<br>• Adherence to treatment: reported for splint group but not control group

Risk of bias

Random sequence generation (selection bias)	Unclear risk of bias	Quote: "randomly assigned"<br>Comment: insufficient information
Allocation concealment (selection bias)	Unclear risk of bias	Quote: "randomly assigned"<br>Comment: insufficient information
Blinding of participants and personnel (performance bias)	High risk of bias	Blinding not possible
Blinding of outcome assessment (detection bias)	High risk of bias	Subjective outcomes assessment by patients (except for 'change in restricted mouth opening' which was objective and measured by a blinded assessor)

Incomplete outcome data (attrition bias)	High risk of bias	Overall attrition 6% (Gp A: 12; Gp B: 0%); "subjects who dropped out had lower pain scores and less motivation to participate in treatment" - this may have biased the results
Selective reporting (reporting bias)	High risk of boas	Outcomes poorly reported and mostly unusable
Other bias	Low risk of bias	No other bias apparent

**Wright 1995** [32]	
**Characteristics**	
Study details	**Trial design:** parallel (3 arms)<br>**Location:** TMJ and Craniofacial Pain Clinic, University of Minnesota, USA<br>**Number of centres:** 1<br>**Recruitment period:** not reported<br>**Sample size calculation:** not reported<br>**Funding:** not reported<br>**Declarations/conflicts of interest:** not reported
Participants	**Diagnosis:** orofacial pain with clinical evidence of a masticatory muscle origin (medical history and clinical examination used to rule out other sources of pain such as dental, metabolic and neurologic disorders); inclusion criteria included: 1) patient's pain aggravated by jaw function (e.g. talking/eating) or parafunctional habits (e.g. clenching or grinding teeth) – based on patient history, 2) pain aggravated/duplicated by palpation of the muscles of mastication – based on clinical examination; TMJ intra-articular sources of pain ruled out by exclusion criteria: 1) pain aggravated by clinical loading of TMJ – based on clinical examination, 2) pain aggravated by TMJ clicking or catching or both – based on patient history and clinical examination<br>**Duration since presenting condition began:** not reported

	**Age at baseline (years):** (overall range 19 to 51): Gp A: mean 34; Gp B: mean 31<br>**Gender:** not reported<br>**Number randomised:** 20 (Gp A: 10; Gp B: 10)<br>**Number evaluated:** 20 (Gp A: 10; Gp B: 10)
Interventions	**Comparison:** splint vs no splint for TMD<br>**Gp A:**<br>• Splint type: custom soft splint<br>• Lower jaw<br>• Material: soft (3.8 mm-thick resilient mouth guard material - Dentiform)<br>• Teeth coverage: full<br>• Details of impression taking: not reported<br>• Instructions to patients: wear all day except when eating meals<br>• Monitoring of patients: not reported<br>**Gp B:** no treatment<br>**Gp C:** palliative treatment (verbal and written instructions on self-care: applying moist heat or ice, eating soft diet, decreasing oral parafunctional habits, decreasing caffeine, modifying sleeping posture, using over-the-counter medication) (not used due to more appropriate control group consisting of no treatment)<br>**Duration of treatment:** Gp A: mean 6.3 weeks; Gp B: mean 6.7 weeks (range 4 to 11 weeks)
Outcomes	Assessed at end of treatment (roughly 6 weeks): grouped under 0 to 3 month analysis)<br>**Primary:**<br>• Pain: muscle pain threshold assessed with a pressure algometer on the anterior temporal muscle and on the superior and inferior areas of the masseter muscle (psi)<br>• Harms/adverse effects: occlusal contact changes<br>**Secondary:**<br>• Change in restricted mouth opening: maximum pain-free opening (from incisor to incisor in mm)

Risk of bias

Random sequence	Low risk of bias	1) "Randomization was made in blocks to maintain equal group sizes" and 2) "two additional subjects were sequentially added to the study and assigned to the

generation (selection bias)		groups in the order that the dropouts were originally assigned"<br>Comments: 1) probably done, 2) unlikely to affect the results in any meaningful way
Allocation concealment (selection bias)	Unclear risk of bias	"Randomization was made in blocks to maintain equal group sizes"<br>Comments: insufficient information
Blinding of participants and personnel (performance bias)	High risk of bias	Blinding not possible
Blinding of outcome assessment (detection bias)	High risk of bias	"final evaluations were with the same independent, blinded examiner who performed the initial evaluation"<br>Comment: although a blinded examiner carried out the pain assessment procedure, the patient was not blinded and this could introduce bias
Incomplete outcome data (attrition bias)	Low risk of bias	Two drop-outs but they were replaced (see above)
Selective reporting (reporting bias)	Low risk of bias	Outcomes fully reported
Other bias	Low risk f bias	No other apparent bias

**Yu 2016** [27]	
**Characteristics**	
Study details	**Trial design:** parallel (4 arms)<br>**Location:** Department of Prosthodontics, Shanghai Ninth People's Hospital, Shanghai, China<br>**Number of centres:** 1<br>**Recruitment period:** February 2013 to March 2015

	**Sample size calculation:** not reported<br>**Funding:** unclear if public or other (Fund of Construction of Shanghai Key Subject, T0202)<br>**Declarations/conflicts of interest:** not reported
Participants	**Diagnosis:** TMJ disc displacement without reduction (RDC/TMD)<br>**Duration since presenting condition began:** unclear<br>**Age at baseline (years):** mean 32.5 (SD 9.8) (only overall data available)<br>**Gender:** 11.3% male (only overall data available)<br>**Number randomised:** 168 (Gp A: 42; Gp B: 42; Gp C: 42; Gp D: 42)<br>**Number evaluated:** 168 (Gp A: 42; Gp B: 42; Gp C: 42; Gp D: 42)
Interventions	**Comparison:** splint vs no/minimal treatment for TMD<br>We split the four groups/arms into two pairwise comparisons of A vs D and C vs B<br>**Gp A:**<br>• Splint type: custom stabilised (Michigan) splint<br>• Upper jaw<br>• Material: transparent base resin<br>• Teeth coverage: full<br>• Details of impression taking: alginate was used to take the impression of both upper and lower dentitions, wax and the "chin point guided CR position" method were used to record patients' centric relation position<br>• Instructions to patients: 20h/day usage<br>• Monitoring of patients: not reported<br>**Gp B:** Manipulative and physical therapies (MPT)<br>• Manipulative therapy: application of the proprioception neuromuscular promoting technique and joint mobilization<br>• Physical therapy: ultra-short wave therapy and ultrasonic therapy<br>**Gp C:** stabilised splint therapy plus MPT (see the above)<br>**Gp D:** control (TMJ related health instructions)<br>**Duration of treatment:** 3 months
Outcomes	Assessed at 3 months: grouped under 0 to 3 month analysis<br>**Primary:**<br>• Pain: current pain intensity - spontaneous masseter pain, palpation pain and chewing pain were separately measured, using a 0 to 10 VAS card made by the Chinese Medical Association (we used spontaneous masseter pain as it is most

	comparable with other included studies; we converted the scale to 0 to 100)<br>**Secondary:**<br>• Change in restricted mouth opening: pain free unassisted maximum mouth opening	
**Risk of bias**		
Random sequence generation (selection bias)	Unclear risk of bias	"Patients were randomly allocated to four groups"<br>Comment: insufficient information
Allocation concealment (selection bias)	Unclear risk of bias	"Patients were randomly allocated to four groups"<br>Comment: insufficient information
Blinding of participants and personnel (performance bias)	High risk of bias	Blinding not possible
Blinding of outcome assessment (detection bias)	High risk of bias	Pain assessed by patients, who were not blinded
Incomplete outcome data (attrition bias)	Low risk of bias	No drop-outs
Selective reporting (reporting bias)	Low risk of bias	Outcomes fully reported
Other bias	Low risk of bias	No other apparent bias
**Zuim 2006** [44]		
**Characteristics**		
Study details	**Trial design:** parallel (4 arms)	

	**Location:** Temporomandibular Disorders Diagnostic and Treatment Centre, Aracatuba Dental School, Sao Paulo State University, Brazil<br>**Number of centres:** 1<br>**Recruitment period:** not reported<br>**Sample size calculation:** not reported<br>**Funding:** not reported<br>**Declarations/conflicts of interest:** not reported
Participants	**Diagnosis:** TMD patients with chronic pain, muscle pain on palpation<br>**Duration since presenting condition began:** at least 6 months<br>**Age at baseline (years):** 13 to 47 (not reported by group)<br>**Gender:** 10% male (not reported by group)<br>**Number randomised:** 20 (Gp A: 5; Gp B: 5; Gp C: 5; Gp D: 5)<br>**Number evaluated:** 20 (Gp A: 5; Gp B: 5; Gp C: 5; Gp D: 5)
Interventions	**Comparison:** splint vs no splint for TMD<br>We split the four groups/arms into two pairwise comparisons of A vs B and C vs D:<br>• Groups A and B had microcurrent electrical nerve stimulation (MENS) on affected muscles using conductive pads or probes; eight applications of 10 minutes each (twice per week over 4 weeks)<br>• Groups C and D had placebo MENS (apparatus was turned off)<br>**Gp A:**<br>• Splint type: occlusal splint<br>• Upper jaw<br>• Material: hard (heat cured acrylic resin)<br>• Teeth coverage: full<br>• Details of impression taking: maxillary and mandibular alginate impressions taken; impressions were poured using special gypsum type IV and the casts were mounted in semi-adjustable articulator<br>• Instructions to patients: not reported<br>• Monitoring of patients: evaluated at weekly intervals for necessary adjustments<br>**Gp B:** no other treatment<br>**Gp C:** same splint as Gp A<br>**Gp D:** no other treatment<br>**Duration of treatment:** 1 month

Outcomes	Assessed at 1 month: we would have grouped under 0 to 3 month analysis<br>**Primary:**<br>• Pain: 0 (no pain) to 10 (worst pain) cm VAS (not clear if current/worst/average) (no usable data - IPD but only 5 pts per group)	
**Risk of bias**		
Random sequence generation (selection bias)	Unclear risk of bias	"the patients were randomly placed in one of four treatment modalities"<br>Comment: insufficient information
Allocation concealment (selection bias)	Unclear risk of bias	"the patients were randomly placed in one of four treatment modalities"<br>Comment: insufficient information
Blinding of participants and personnel (performance bias)	High risk of bias	Blinding not possible
Blinding of outcome assessment (detection bias)	High risk of bias	Pain assessed by patients, who were not blinded
Incomplete outcome data (attrition bias)	Low risk of bias	All randomised patients were included in the analyses
Selective reporting (reporting bias)	Low risk of bias	Individual patient data reported
Other bias	Low risk of bias	No other apparent bias

Supplementary Appendix 3: Risk of bias summary

	Random sequence generation (selection bias)	Allocation concealment (selection bias)	Blinding of participants and personnel (performance bias)	Blinding of outcome assessment (detection bias)	Incomplete outcome data (attrition bias)	Selective reporting (reporting bias)	Other bias
Conti 2005	?	?	−	−	?	−	?
Conti 2012	?	?	−	−	−	−	?
Conti 2015	?	?	−	−	−	−	?
Costa 2015	+	+	−	−	−	−	+
Daif 2012	+	?	−	−	+	−	+
de Felicio 2006	+	?	−	−	+	−	+
de Felicio 2010	+	?	−	−	?	+	+
DeVocht 2013	+	+	−	−	+	+	+
Elsharkawy 1995	?	?	−	−	−	−	+
Ficnar 2013	?	?	−	−	+	+	+
Giannakopoulos 2016	+	+	−	−	+	+	+
Gomes 2014	+	+	−	+	+	−	+
Gomes 2015	+	+	−	−	−	−	+
Haketa 2010	+	+	−	−	+	+	+
Hasanoglu 2017	?	?	−	−	+	+	+
Johansson 1991	?	?	−	−	+	+	−
Katyayan 2014	+	+	−	−	+	+	+
Leeson 2007	+	+	−	−	+	+	+
List 1992	?	?	−	−	+	−	−
Lundh 1985	?	?	−	−	+	−	+
Lundh 1988	?	?	−	−	+	−	+
Lundh 1992	?	?	−	−	+	−	+
Magnusson 1999	?	?	−	−	+	+	+
Michelotti 2012	+	?	−	−	+	+	+
Nagata 2015	+	?	−	−	+	+	+
Niemela 2012	+	?	−	−	+	+	+
Nitecka-Buchta 2014	+	+	−	−	−	+	+
Pierce 1988	?	?	−	+	?	−	+
Rampello 2013	?	?	−	−	+	+	−
Sharma 2016	+	?	−	−	?	+	+
Tatli 2017	+	?	−	−	+	+	+
Tavera 2012	?	?	−	−	−	−	+
Truelove 2006	+	+	−	−	−	+	+
Wahlund 2003	?	?	−	−	−	−	+
Wright 1995	+	?	−	−	+	+	+
Yu 2016	?	?	−	−	+	+	+
Zuim 2006	?	?	−	−	+	+	+

Supplementary Appendix 4: Summary effect estimates for outcomes other than pain for splints versus no/minimal intervention in TMD patients

Outcome	Studies (Participants)	Effect Estimate (95%CI) (Random Effects)	P-value for effect estimate	Heterogeneity	
				Chi-Square P-value	I^2
TMJ clicking: presence of joint noises (detected during TMJ palpation /opening/closing) - 0 to 3 months	3 (252); 5 pairwise comparisons	RR 0.85 [0.51, 1.43]	0.55	0.001	77%
>3 to 6 months	3 (131); 4 pairwise comparisons	RR 0.90 [0.79, 1.03]	0.13	0.76	0%
>6 to 12 months	2 (238); 4 pairwise comparisons	RR 0.90 [0.74, 1.10]	0.30	0.15	43%
Change in restricted mouth opening: maximum mouth opening (mm) - 0 to 3 months	13 (913); 16 pairwise comparisons	MD (mm) 1.17 [-0.68, 3.03]	0.22	<0.00001	83%
>3 to 6 months	3 (236)	MD (mm) 0.29 [-0.63, 1.20]	0.54	0.30	18%

Quality of life: Oral Health Impact Profile (OHIP-14) 0 to 56 worsening scale - 0 to 3 months	2 (80)	MD -1.43 [-5.11, 2.24]	0.44	0.62	0%
>3 to 6 months	2 (76)	MD 0.90 [-3.94, 5.74]	0.72	0.21	36%
>6 to 12 months	1 (43)	MD 1.31 [-5.11, 7.73]	0.69	N/A	N/A

Supplementary Appendix 5: Summary of findings table

Oral splints for patients with orofacial signs or symptoms to reduce orofacial pain						
Patient or population: patients provided with oral splints for TMD<br><br>Setting: primary or secondary care<br><br>Intervention: oral splint<br><br>Comparison: no splint/minimal intervention						
Outcomes	Illustrative comparative risks (95%CI)		Relative effect (95%CI)	Number of participants (studies)	Certainty of the evidence (GRADE)	Comments
	Assumed risk	Corresponding risk				
	No splint	Oral splint				
Pain SD units:<br><br>Pain measured on combinable scale	The pain score in the oral splint group was on average 0.18 SDs lower (0.06 higher to 0.42 lower) than the no/minimal intervention group			1076<br>(13 RCTs; 16 pairwise comparisons)	⊕⊖⊖⊖<br>very low [1]	No evidence that splints reduced pain.

0 to 3 months<br><br>(unable to use MD due to differences in the way pain was measured in the studies)						As rule of thumb, 0.2 SD represents a small difference, 0.5 a moderate difference and 0.8 a large difference.<br><br>Similar effect sizes at other time points.
Current pain intensity measured on VAS (0 to 100 mm) or NRS 0 to 100<br><br>At 0 to 3 months	The mean pain intensity in the control groups ranged from 9.23 to 41.1 mm[3], median = 20	The mean pain intensity in the splint groups was 4.48 mm lower (11.59 lower to 2.64 higher)		874<br>(11 RCTs; 13 pairwise comparisons)	⊕⊖⊖⊖<br>very low [1]	Results similar at other time points

Clicking of joint at 0 to 3 months (Yes/No)	500[2] per 1000	425 per 1000 (255 to 715)	RR 0.85 [0.51, 1.43]	252 (3 RCTs; 5 pairwise comparisons)	⊕⊖⊖⊖ very low [1]	No evidence of a difference in joint clicking.<br>Results similar at other time points.
Maximum mouth opening at 0 to 3 months (mm)	The mean maximum mouth opening in the control groups ranged[3] from 33.08 to 47.1 mm, median 40 mm	The mean maximum mouth opening in the splint groups was 1.17 mm higher (0.68 lower to 3.03 higher)		913 (13 RCTs; 16 pairwise comparisons)	⊕⊖⊖⊖ very low [1]	No evidence of a difference in maximum mouth opening.<br>The results at >3 to 6 months MD (mm) 0.29 [-0.63, 1.20]. No data at >6 to 12 months.
Quality of life using Oral Health Impact Profile (OHIP-14) 0 to 56	The mean[4] score in the control groups was 14.84	The mean score in the splint groups was 1.43 lower (5.11 lower to 2.24 higher)		80 (2 RCTs)	⊕⊖⊖⊖ very low [1]	No evidence of a difference in quality of life.

worsening scale at 0 to 3 months						Similar results at other time points.
Adverse events	None of the studies reported any adverse events					
**GRADE Working Group grades of evidence**<br>**High certainty:** We are very confident that the true effect lies close to that of the estimate of the effect<br>**Moderate certainty:** We are moderately confident in the effect estimate: The true effect is likely to be close to the estimate of the effect, but there is a possibility that it is substantially different<br>**Low certainty:** Our confidence in the effect estimate is limited: The true effect may be substantially different from the estimate of the effect<br>**Very low certainty:** We have very little confidence in the effect estimate: The true effect is likely to be substantially different from the estimate of effect						

Footnotes

[1] Downgraded as all studies at high risk of bias, substantial heterogeneity, lack of precision

[2] Median event rate for no/minimal intervention group

[3] Range does not include two studies that reported change scores

[4] This is the mean in the study that reported an end score, as the other study reported a change score
